# Disrupted multi-scale topological organization of directed functional brain networks in patients with disorders of consciousness

**DOI:** 10.1093/braincomms/fcad069

**Published:** 2023-03-28

**Authors:** Yu Guo, Bolin Cao, Yanbin He, Qiuyou Xie, Qimei Liang, Yue Lan, Mingxian Zhang, Yidan Qiu, Ronghao Yu, Ruiwang Huang

**Affiliations:** School of Psychology, Center for Studies of Psychological Application, Guangdong Key Laboratory of Mental Health and Cognitive Science, Ministry of Education Key Laboratory of Brain Cognition and Educational Science, South China Normal University, Guangzhou, Guangdong, 510631, China; School of Psychology, Center for Studies of Psychological Application, Guangdong Key Laboratory of Mental Health and Cognitive Science, Ministry of Education Key Laboratory of Brain Cognition and Educational Science, South China Normal University, Guangzhou, Guangdong, 510631, China; Traumatic Brain Injury Rehabilitation Department & Severe Rehabilitation Department, Guangdong Province Work Injury Rehabilitation Hospital, Guangzhou, Guangdong, 510440, China; Department of Rehabilitation Medicine, Zhujiang Hospital of Southern Medical University, Guangzhou, Guangdong, 510280, China; Department of Rehabilitation Medicine, Zhujiang Hospital of Southern Medical University, Guangzhou, Guangdong, 510280, China; Department of Rehabilitation Medicine, Zhujiang Hospital of Southern Medical University, Guangzhou, Guangdong, 510280, China; School of Psychology, Center for Studies of Psychological Application, Guangdong Key Laboratory of Mental Health and Cognitive Science, Ministry of Education Key Laboratory of Brain Cognition and Educational Science, South China Normal University, Guangzhou, Guangdong, 510631, China; School of Psychology, Center for Studies of Psychological Application, Guangdong Key Laboratory of Mental Health and Cognitive Science, Ministry of Education Key Laboratory of Brain Cognition and Educational Science, South China Normal University, Guangzhou, Guangdong, 510631, China; Cihui Hospital, Guangzhou, Guangdong, 510645, China; School of Psychology, Center for Studies of Psychological Application, Guangdong Key Laboratory of Mental Health and Cognitive Science, Ministry of Education Key Laboratory of Brain Cognition and Educational Science, South China Normal University, Guangzhou, Guangdong, 510631, China

**Keywords:** disorders of consciousness, time delay estimation, degree, motif, canonical correlation analysis

## Abstract

Disorders of consciousness are impaired states of consciousness caused by severe brain injuries. Previous resting-state functional magnetic resonance imaging studies have reported abnormal brain network properties at different topological scales in patients with disorders of consciousness by using graph theoretical analysis. However, it is still unclear how inter-regional directed propagation activities affect the topological organization of functional brain networks in patients with disorders of consciousness. To reveal the altered topological organization in patients with disorders of consciousness, we constructed whole-brain directed functional networks by combining functional connectivity analysis and time delay estimation. Then we performed graph theoretical analysis based on the directed functional brain networks at three topological scales, from the nodal scale, the resting-state network scale to the global scale. Finally, the canonical correlation analysis was used to determine the correlations between altered topological properties and clinical scores in patients with disorders of consciousness. At the nodal scale, we observed decreased in-degree and increased out-degree in the precuneus in patients with disorders of consciousness. At the resting-state network scale, the patients with disorders of consciousness showed reorganized motif patterns within the default mode network and between the default mode network and other resting-state networks. At the global scale, we found a lower global clustering coefficient in the patients with disorders of consciousness than in the controls. The results of the canonical correlation analysis showed that the abnormal degree and the disrupted motif were significantly correlated with the clinical scores of the patients with disorders of consciousness. Our findings showed that consciousness impairment can be revealed by abnormal directed connection patterns at multiple topological scales in the whole brain, and the disrupted directed connection patterns may serve as clinical biomarkers to assess the dysfunction of patients with disorders of consciousness.

## Teaser

Guo *et al*. reported the abnormal connection patterns based on directed functional brain networks at multiple topological scales and their correlations with clinical performances for the patients with disorders of consciousness. The findings suggested that directed interactions at multiple scales may exert a crucial role in the maintenance of consciousness.

## Graphical Abstract

**Graphical abstract:**
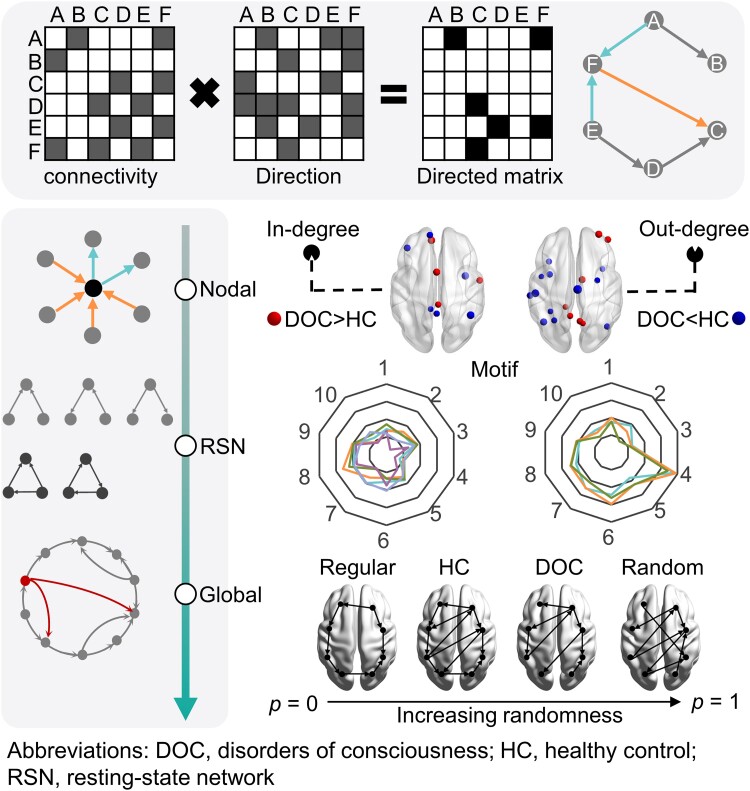

## Introduction

Disorders of consciousness (DOC) are caused by severe brain injuries and include the vegetative state/unresponsive wakefulness syndrome (VS/UWS) and the minimally conscious state (MCS). VS is characterized by no behavioural signs of self-related or environmental awareness,^1^ and MCS is recognized by minimal, inconsistent, but reproducible signs of awareness.^2^ The understanding of neuropathology on impaired consciousness has greatly benefited from detecting the disorganization of functional brain networks in DOC patients based on functional magnetic resonance imaging (fMRI) data.^3,4^

Functional brain networks are organized across multiple topological scales ranging from individual regions (nodes) to the resting-state networks (RSNs) and the whole brain.^5^ Graph theoretical analysis can be used to detect the altered topological organization in large-scale brain networks for DOC patients by measuring the inter-regional connection patterns.^6^ Previous DOC patients’ studies have reported abnormal connection patterns in the precuneus,^7‐9^ and prominent disorganization within and between the default mode network (DMN) and the frontoparietal network (FPN).^10‐13^ The graph theoretical analysis across multiple scales can provide detailed information on the disrupted connection patterns of functional brain networks for DOC patients.^14^

However, the majority of network studies for DOC patients relied on functional connectivity (FC) analysis measuring static relationships of inter-regional time series, which is technically straightforward but neglects the direction of interaction between distinct brain regions. Detecting the topological organization of directed functional brain networks may reveal the neural mechanism underlying impaired consciousness in DOC patients.^15^ Dynamic causal modelling (DCM) is a common method to infer a directed functional connection.^16^ Using this method, previous studies have found abnormal inter-regional interactions in DOC patients.^17‐20^ However, the DCM is based on specific hypotheses and limited in investigating the connection patterns of several brain regions, which is unsuitable for constructing a large-scale graph. Time delay estimation (TDE), a method recently used for calculating directed propagation activities, does not impose a constraint on the topological scale and could be applied in the whole-brain analysis.^21^ TDE has been widely used in revealing the characteristic of directed brain networks in healthy populations^22‐25^ and altered directed interactions in brain disorders.^26‐29^

The current study aims to construct whole-brain directed functional networks by combining FC analysis and TDE, and perform the graph theoretical analysis at multiple topological scales to detect the abnormal brain network organization in DOC patients. Specifically, we estimated topological properties at the nodal scale, the RSN scale and the global scale in directed functional brain networks. At the nodal scale, the nodal degree and the hub disruption index were calculated to identify the regions with abnormal directed connection patterns in DOC patients. At the RSN scale, we used motif to detect the aberrant organization rules within and between RSNs in DOC patients. At the global scale, the global efficiency and the global clustering coefficient were computed to describe the alterations of whole-brain functional integration and segregation in DOC patients. Finally, we performed a canonical correlation analysis (CCA) to reveal the relationships between the abnormal directed graph theoretical metrics and clinical scores in DOC patients. We hypothesized that DOC was associated with the breakdown of directed interactions at multiple topological scales in functional brain networks.

## Materials and methods

### Participants

A total of 45 DOC patients were recruited from the Liuhuaqiao Hospital in Guangzhou City, Guangdong Province, China. We excluded low-quality resting-state fMRI (rs-fMRI) data using the following exclusion criteria: (1) severe brain atrophy or focal brain damage (12 patients), (2) artefacts (4 patients) or (3) excessive head motion (translation ≥ 3 mm in any plane or rotation ≥ 3° in any axis) during the rs-fMRI scanning (8 patients). In the end, we included a total of 21 DOC patients (17M/4F, aged 36.95 ± 13.59 years old) for the subsequent analyses. The Coma Recovery Scale-Revised (CRS-R) was used to assess the states of consciousness of the DOC patients by two medical doctors (QX and RY). CRS-R is a 23-item scale for measuring the behavioural responses of DOC patients, which includes 6 sub-scales for measuring auditory, visual, motor, oromotor, communication and arousal levels, respectively. The detailed clinical information for these patients is listed in Table 1. In addition, we also recruited 21 healthy subjects as the controls (12M/9F, aged 31.38 ± 7.67 years old). None of them had a history of neurological or psychiatric disorders. This study was approved by the Institute Review Board (IRB) of the Liuhuaqiao Hospital. Written informed consent was obtained from the healthy subjects or the legal surrogates of the patients prior to the study.

**Table 1:** Demographic and clinical information for the patients with DOC

Patient	Sex	Months from injury to scan	Age (years old)	VS/MCS	Aetiology	CRS-R scores
						Aud/Vis/Mot/Oro/Com/Aro/T
P01	F	1	28	VS	HIE	1/0/2/1/0/2/6
P02	M	1	28	VS	HIE	1/0/2/0/0/2/5
P03	M	2	21	VS	HIE	1/0/2/1/0/1/5
P04	M	3	39	VS	TBI	0/0/2/1/0/2/5
P05	M	2	16	VS	HIE	0/0/1/0/0/2/3
P06	M	1	64	VS	TBI	0/0/1/1/0/2/4
P07	M	1	62	VS	HIE	0/0/1/0/2/2/5
P08	M	1	43	VS	HIE	0/0/1/1/0/2/4
P09	M	1	48	VS	HIE	0/0/1/0/0/2/3
P10	M	9	32	VS	HIE	1/0/1/1/0/2/5
P11	M	1	39	VS	HIE	0/0/2/1/0/2/5
P12	M	1	36	VS	TBI	0/0/1/2/0/2/5
P13	M	2	36	VS	HIE	1/0/0/0/0/1/2
P14	M	2	47	VS	TBI	1/3/0/1/0/2/7
P15	M	1	30	MCS	TBI	1/1/3/2/0/2/9
P16	F	1	59	MCS	TBI	1/0/5/1/0/2/9
P17	F	1	15	MCS	HIE	1/3/5/1/0/2/12
P18	F	2	20	MCS	TBI	1/1/3/1/0/2/8
P19	M	1	41	MCS	HIE	2/3/2/1/0/1/9
P20	M	1	31	MCS	TBI	1/4/5/2/2/3/17
P21	M	1	41	MCS	TBI	1/1/3/0/0/2/7

M, male; F, female; VS, vegetable state; MCS, minimally conscious state; TBI, traumatic brain injury; HIE, hypoxic ischemic encephalopathy; CRS-R, Coma Recovery Scale-Revised. Aud, auditory; Vis, visual; Mot, motor; Oro, oromotor; Com, communication; Aro, arousal; T, total CRS-R score.

### Data acquisition

All MRI data were acquired on a GE Signa HDX 3 T MR scanner with an eight-channel phased-array head coil. The rs-fMRI data were obtained using a single-shot multi-slice gradient-echo EPI sequence with the following parameters: repetition time (TR) = 2000 ms, echo time (TE) = 26 ms, flip angle (FA) = 90°, field of view (FOV) = 240 × 240 mm^2^, data matrix = 64 × 64, slice thickness = 3.6 mm, inter-slice gap = 0.6 mm, 36 axial interleaved slices covering the whole brain and 240 volumes obtained in about 8 min. The high-resolution brain structural images were obtained using a T_1_-weighted 3D fast spoiled gradient recalled sequence with the following parameters: TR = 8.86 ms, TE = 3.52 ms, FOV = 240 × 240 mm^2^, data matrix = 256 × 256, FA = 90°, voxel size = 0.94 × 0.94 × 1  mm^3^ and 176 sagittal slices. Both the fMRI and structural data were acquired in the same session for each subject.

### Preprocessing of rs-fMRI data

The rs-fMRI data were preprocessed using SPM12 (https://www.fil.ion.ucl.ac.uk/spm) and DPABI (http://www.rfmri.org/dpabi).^30^ Specifically, we performed (1) removal of the first 10 time points, (2) slice-timing correction, (3) head-motion correction, (4) normalization of the functional and structural images into the MNI-152 standard space with a 3 mm^3^ isotropic resolution, (5) spatial smoothing with a 6 mm full width at half-maximum Gaussian kernel and (6) band-pass filtering (0.01–0.1 Hz). In addition, we regressed out the nuisance variables, including 24 head movement parameters,^31^ global brain signal, white matter signal and cerebrospinal fluid signal.

### Construction of directed functional brain networks

For each subject, we parcellated the brain into 264 functional regions of interest (ROIs) according to the Power-264 template.^32^ Each ROI was delineated as a sphere of 5 mm radius centred on the peak coordinate. The Power-264 template provides a mechanism to localize ROIs to specific RSNs^33^ and has been widely used in neuroimaging studies of healthy participants^33,34^ and various patients, including DOC patients ^8,13,35^ and other neurological or psychiatric populations.^36‐39^ We first excluded 37 ROIs that did not match any established RSN.^32,40,41^ The retained 227 ROIs, including 214 cortical and 13 subcortical ROIs, were assigned to the following 10 RSNs: sensorimotor network (35 ROIs), cingulo-opercular network (CON, 14 ROIs), auditory network (AUN, 13 ROIs), DMN (58 ROIs), visual network (VSN, 31 ROIs), FPN (25 ROIs), salience network (SAN, 18 ROIs), subcortical network (SUN, 13 ROIs), ventral attention network (9 ROIs) and dorsal attention network (DAN, 11 ROIs). In this study, we took each ROI as a node and the inter-nodal directed functional connection as an edge, which was determined by combining the FC analysis with the TDE as follows.

To represent the strength of the edge, we derived the FC matrix for each subject by calculating Pearson’s correction coefficient between all pairs of time series. We only retained positive correlation values in each FC matrix because the interpretation of negative correlation is ambiguous.^42,43^ Then, according to the method applied in previous DOC studies,^11,44,45^ we binarized the FC matrix at four different threshold levels (10%, 15%, 20% and 25%). Specifically, for a given threshold, we took the above threshold elements as 1 and the others as 0 for each subject.

**Figure 1:**
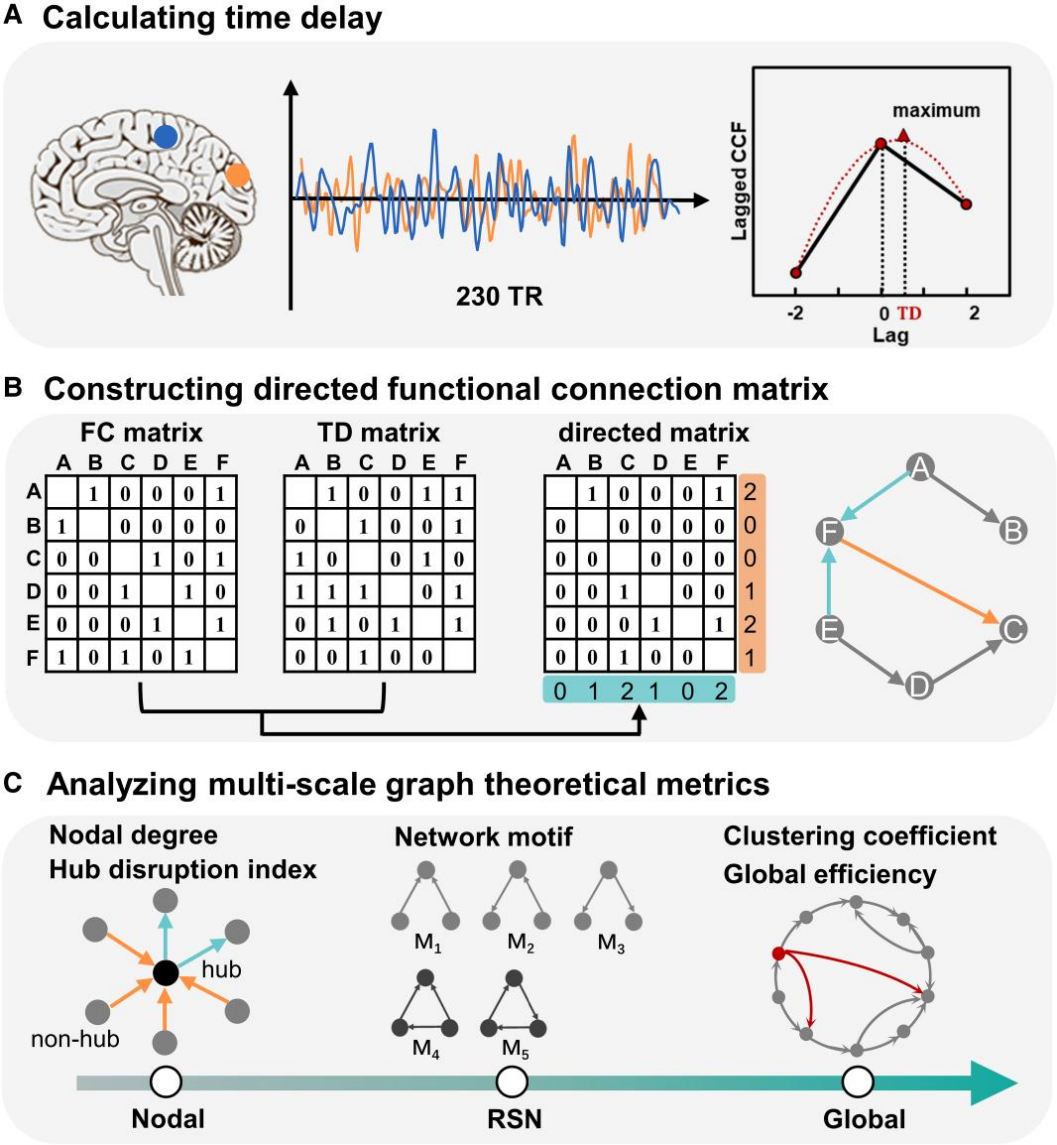
**Flowchart for constructing the directed functional connection matrix and analysing multi-scale graph theoretical metrics.** (**A**) Steps for calculating TD. We first calculated the lagged CCF between any pair of time series at several lag values (*τ* = 0 TR, ± 1 TR, ± 2 TRs and ±3 TRs). Then we adopted a parabolic interpolation to fit the CCF curve and defined the lag corresponding to the maximum of the CCF curve as TD. (**B**) Construction of the directed functional connection matrix. The directed functional connection matrix was obtained by multiplying each element of the FC matrix by the counterpart of the TD matrix for each subject. In the directed matrix, the element with the value 1 represents an edge from the node in a given row to the node in a given column. For example, node F has two incoming edges connecting with nodes A and E and one outgoing edge connecting with node C. (**C**) Analysis of graph theoretical metrics at three topological scales: the nodal scale, the RSN scale and the global scale.

The direction of each edge was determined using TDE. For two time series (*x_i_* and *x_j_*) from two nodes, we fixed one time series and shifted the other forward or backward by several lag values (denoted as τ, 0 TR, 1 TR, 2 TRs and 3 TRs) to calculate the lagged cross-covariance function (CCF). The lagged CCF represents the correlation between two time series at all possible τ and is given by


(1) $$\begin{array}{c} C_{x_{i}x_{j}}(\tau )=\frac{1}{T}\int_{t=1}^{t=230}x_{i}(t+\tau )\cdot x_{j}(t)dt, \end{array}$$

where $x_{i}$ and $x_{j}$ represent the time series of node *i* and node *j*, respectively, and *T* represents the integration interval. Then, we interpolated these discrete lagged CCF values to acquire a smoothed curve. The value corresponding to the maximum lagged CCF in the curve is defined as the time delay (TD*_ij_*) (Fig. 1A). The detailed description and the code for the TDE can be found in Raut *et al*.^46^ According to the TD between all pairs of nodes, we constructed the TD matrix for each subject. Specifically, if TD_*ij*_ > 0, *x_i_* lagged relative to *x*_*j*_, and the value was denoted as 1 representing that the direction of the edge was from node *j* to node *i*, otherwise, we denoted the value as 0. In this way, the edge direction for all pairs of nodes was indicated by the TD matrix.

Finally, for each subject, we constructed the directed functional connection matrix by multiplying each element in the FC matrix by its counterpart in the TD matrix, as illustrated in Fig. 1B. Based on the directed functional connection matrix, we performed the graph theoretical analysis at multiple topological scales (Fig. 1C) which were described in the following subsection. To ensure the robustness of the results, the graph theoretical metrics were averaged across the four thresholds (10%, 15%, 20% and 25% for the FC matrix) for each subject.^11^

### Graph theoretical analysis

#### Nodal degree analysis

At the nodal scale, we calculated in-degree (*D*^in^) and out-degree (*D*^out^)^47^ to measure the driven and driving connection patterns between brain regions. For a given node, the *D*^in^ is the number of incoming edges that point to the node and the *D*^out^ is the number of outgoing edges that point away from the node (Supplementary Materials). The nodes with a high *D*^in^ (*D*^out^) value are considered driven (driving) hubs, which exert more influence over brain network function compared with the nodes with fewer connections. We identified the top 2.5% of the nodes with the highest *D*^in^ (*D*^out^) as the driven (driving) hubs according to a previous study.^48^

In addition, we also calculated the hub disruption index of *D*^in^ and *D*^out^ for each subject, denoted as *κ*^in^ and *κ*^out^, respectively, following the method from Achard *et al*.^7^ This method allows us to quantify the reorganization of network hubs in a subject in reference to the normative topology of the control group. Specifically, *κ*^in^ (*κ*^out^) of a subject is the slope fitted to the linear regression model between *D*^in^ (*D*^out^) of the control group and the difference between *D*^in^ (*D*^out^) of the subject and the control group, which is given by


(2) $$\begin{array}{c} \kappa^{in/out}=\frac{y^{in/out}-b^{in/out}}{x^{in/out}}, \end{array}$$

where *y*^in/out^ represents the difference of the *D*^in^ (*D*^out^) of a subject minus the mean *D*^in^ (*D*^out^) of the control group, *x*^in/out^ represents the mean *D*^in^ (*D*^out^) of the control group and *b*^in/out^ represents the residual of the regression. The *κ*^in^ (*κ*^out^) close to −1 suggests radical reorganization of network hubs of the subject, meaning that the nodes with the highest *D*^in^ (*D*^out^) in the controls show the greatest decrease in the subject, or the nodes with the lowest *D*^in^ (*D*^out^) in the controls show the greatest increase in the subject.^7,8^

#### Motif configuration analysis

Motif is the node-connected subgraph consisting of *M* nodes with at least *M−*1 edges, which is used to reveal basic building blocks in directed brain networks.^49,50^ Given a subgraph size, *M*, there is a constant number of wiring configurations, and these configurations are defined as motif classes. For example, when *M* = 2, 3, 4 and 5, the number of motif classes is 2, 13, 196 and 9364, respectively. In this study, we considered the three-node motif (*M* = 3) that has been identified in human structural and functional brain networks.^51‐53^ For the three-node motif, there are five unidirectional classes and eight reciprocal classes. Only the five unidirectional classes were analysed in this study, which were labelled as M_1_, M_2_, M_3_, M_4_ and M_5_ (as shown in Fig. 1C), including two classes of closed-triangle motif and three classes of open-triangle motif. In classes of closed-triangle motif, three nodes are connected to each other. In classes of open-triangle motif, one node is connected to two other nodes that do not directly connect to each other.

The motif frequency spectrum (MFS) was used to calculate the number of occurrences of each class in a certain network.^50^ To detect the rules governing directed connection patterns across the RSNs, we obtained the MFS of the five classes within each RSN, between pairs of RSNs, and between one-versus-all-other RSNs, respectively (Supplementary Materials). In addition, we also analysed the MFS of each class in the whole-brain network to determine the distribution of five motif classes.

#### Global topology analysis

At the global scale, the global efficiency ^47,54^ and the global clustering coefficient ^55,56^ were calculated to analyse the functional integration and segregation of the directed brain network. The description of these graph theoretical metrics is listed in the Supplementary Materials.

### Canonical correlation analysis

CCA is an approach to modelling the maximal correlation between two high-dimensional multivariate datasets based on a linear combination. In this study, we performed CCA to link the abnormal graph theoretical metrics and six sub-scores of CRS-R for DOC patients. Specifically, the graph theoretical metrics with significant between-group differences at any topological scale were linearly combined to a graph theoretical variate, and six sub-scores of CRS-R were linearly combined to a clinical variate for each subject (Supplementary Materials). Three graph theoretical variates were considered as follows: (1) the degree variate, which is the linear combination of *D*^in^ and *D*^out^ metrics with significant between-group differences, (2) the motif variate, which is the linear combination of MFS metrics with significant between-group differences; and (3) the global variate, which is the global metrics with significant between-group differences. The maximal correlation coefficient between any pair of graph theoretical variate and clinical variate was obtained.

To quantify the contribution of original graph theoretical metrics with significant between-group differences and sub-scores of CRS-R for significant CCA modes, we calculated Pearson’s correlation between the graph theoretical metrics and corresponding graph theoretical variates as well as between the sub-scores of CRS-R and corresponding clinical variates for significant CCA modes. Then, the Pearson correlation analysis was performed between the graph theoretical metrics that were significantly correlated with corresponding graph theoretical variates and the sub-scores of CRS-R that were significantly correlated with corresponding clinical variates.

### Statistical analysis

A non-parametric permutation test with 5000 iterations was used to determine significant differences in the *D*^in^, *D*^out^, *κ*^in^, *κ*^out^, MFS, global efficiency, and global clustering coefficient between the DOC patients and the healthy controls. In the calculations, we regressed out age, sex and mean framewise displacement (FD). False discovery rate (FDR) correction was adopted to control multiple comparisons for the statistical analysis at the nodal scale or RSN scale. Specifically, at the nodal scale, all *P*-values of 227 *D*^in^ (*D*^out^) values were corrected using the FDR correction. At the RSN level, all *P*-values of 245 MFS values, including 165 MFS values between pairs of RSNs, 30 MFS values between one-versus-all-other RSNs and 50 values within RSNs, were also corrected by the FDR method. The significance of the CCA modes was estimated using a non-parametric permutation test with 5000 iterations. The significance threshold was set at *P* < 0.05 for all analyses.

### Robustness analysis

To test the robustness of the observed differences in the graph theoretical metrics between the DOC patients and the healthy controls, we repeated the calculations using two additional processing strategies: (1) without spatial smoothing and (2) using the maximum lagged correlation that corresponds to the maximum lagged CCF as the strength of the edge.

## Results

### Demographic and clinical statistics

No significant difference was found in age, sex or mean FD between the DOC patients and the healthy controls. The statistical information of the patients and the controls is listed in Table 2.

**Table 2:** Demographic and clinical statistics for the patients with DOC and the healthy controls (HC)

Characteristics	DOC	HC	Statistics	*P*-value
Age (years old)	36.9 ± 13.9	31.4 ± 7.9	*t* = 1.60	0.120^a^
Sex (M/F)	17/4	12/9	*χ* ^2^ = 2.09	0.159^b^
Mean FD	0.15 ± 0.15	0.09 ± 0.04	*t* = 1.94	0.064^a^
Months from injury to scan	1.71 ± 1.76			
Aetiology (HIE/TBI)	11/9			
Diagnosis (VS/MCS)	14/7			

M, male; F, female; FD, framewise displacement; HIE, hypoxic ischemic encephalopathy; TBI, traumatic brain injury; VS, vegetable state; MCS, minimally conscious state.

a Two-sample *t*-test.

b *χ* ^2^ test.

### Nodal degree analysis

**Figure 2:**
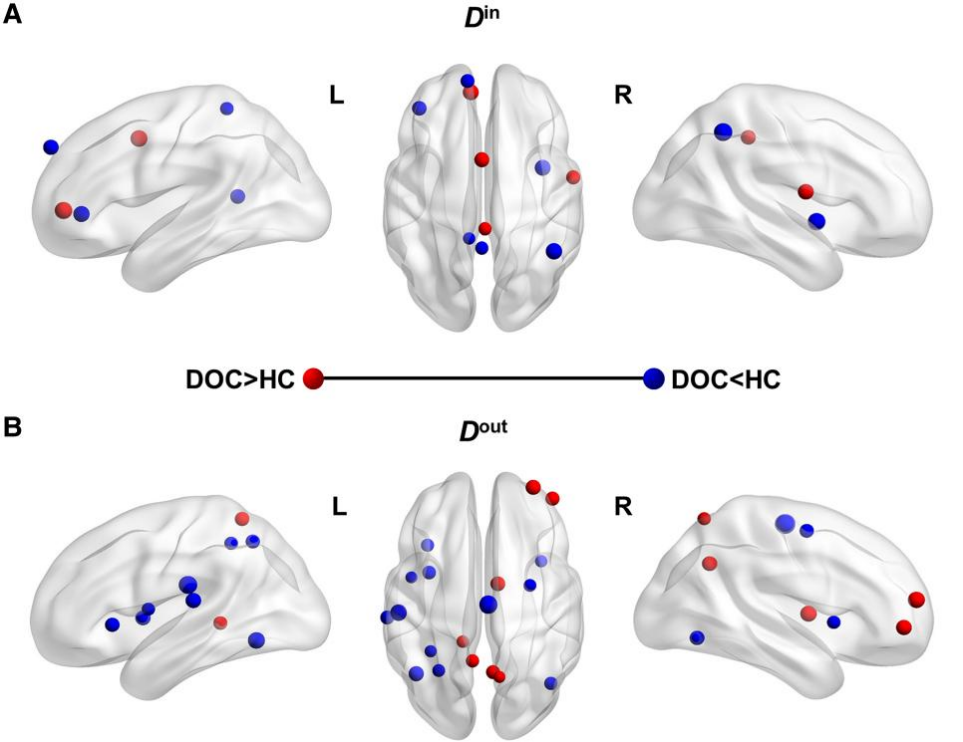
**Nodes with significant differences in either in-degree (*D*^in^) or out-degree (*D*^out^) between the patients with DOC (*n* = 21) and the healthy controls (HC, *n* = 21) (*P* < 0.05, permutation test, FDR-corrected).** (**A**) *D*^in^ and (**B**) *D*^out^. The nodes colour-coded in red (blue) indicate that *D*^in^ or *D*^out^ was significantly higher (lower) in the patients than in the controls. The size of a node is proportional to the absolute value of the *t*-value. The detailed information for these nodes is listed in Table 3.

Figure 2 shows the nodes with significant between-group differences in either *D*^in^ or *D*^out^. The DOC patients showed significantly lower *D*^in^ in six nodes and significantly higher *D*^in^ in four nodes than in the healthy controls. We also found that the *D*^out^ was significantly lower in 12 nodes and significantly higher in 7 nodes in the patients than in the controls. The detailed information for these nodes is listed in Table 3.

**Table 3:** Nodes with significant differences in either in-degree (*D*^in^) or out-degree (*D*^out^) between the patients with DOC and the healthy controls (*P* < 0.05, permutation test, FDR-corrected)

Degree	Node	RSN	Coordinates in MNI space			*t*-Value
			*x*	*y*	*z*	
*D* ^in^	Frontal_Sup_L	DMN	−10	55	39	−3.62
	Cingulum_Mid_L	DMN	0	−37	44	3.49
	Frontal_Sup_Medial_L	DMN	−8	48	3	4.13
	Precuneus_L	DMN	−1	−49	11	−3.50
	Parietal_Inf_R	FPN	44	−51	47	−4.26
	Frontal_Mid_L	FPN	−40	38	1	−3.79
	Frontal_Sup_Medial_L	FPN	−1	6	44	3.95
	Insula_R	CON	37	1	−4	−4.14
	Precuneus_L	SMN	−9	−43	61	−3.21
	Rolandic_Oper_R	AUN	56	−5	13	3.69
*D* ^out^	Temporal_Inf_L	DAN	−42	−60	−9	−3.91
	Parietal_Inf_L	DAN	−33	−46	47	−3.17
	Precentral_R	DAN	29	−5	54	−3.34
	Precuneus_R	DAN	10	−62	61	3.11
	Rolandic_Oper_L	CON	−45	0	9	−3.10
	Insula_L	CON	−34	3	4	−3.57
	Insula_R	CON	36	10	1	−3.20
	Supp_Motor_Area_R	SMN	3	−17	58	−4.79
	Precuneus_L	SMN	−7	−52	61	3.44
	SupraMarginal_L	AUN	−53	−22	23	−4.30
	Temporal_Sup_L	AUN	−60	−25	14	−3.63
	Lingual_L	DMN	−13	−40	1	3.28
	Precuneus_R	DMN	6	−59	35	3.61
	Parietal_Sup_L	FPN	−28	−58	48	−3.34
	Frontal_Inf_Orb_R	FPN	43	49	−2	3.67
	Thalamus_R	SUN	9	−4	6	3.84
	Insula_L	SAN	−35	20	0	−3.42
	Frontal_Sup_R	SAN	31	56	14	3.94
	Temporal_Inf_R	VSN	42	−66	−8	−3.46

*Note.* A positive (negative) *t*-value indicates that *D*^in^ or *D*^out^ was significantly higher (lower) in the patients than in the controls. These nodes are also indicated in Fig. 2.

MNI, Montreal Neurological Institute; RSN, resting-state network; SMN, sensorimotor network; CON, cingulo-opercular network; AUN, auditory network; DMN, default mode network; VSN, visual network; FPN, frontoparietal network; SAN, salience network; SUN, subcortical network; DAN, dorsal attention network; Sup, superior; Inf, inferior; Mid, middle; Oper, operculum; Supp, supplementary; Orb, orbital; L (R), left (right) hemisphere.

**Figure 3:**
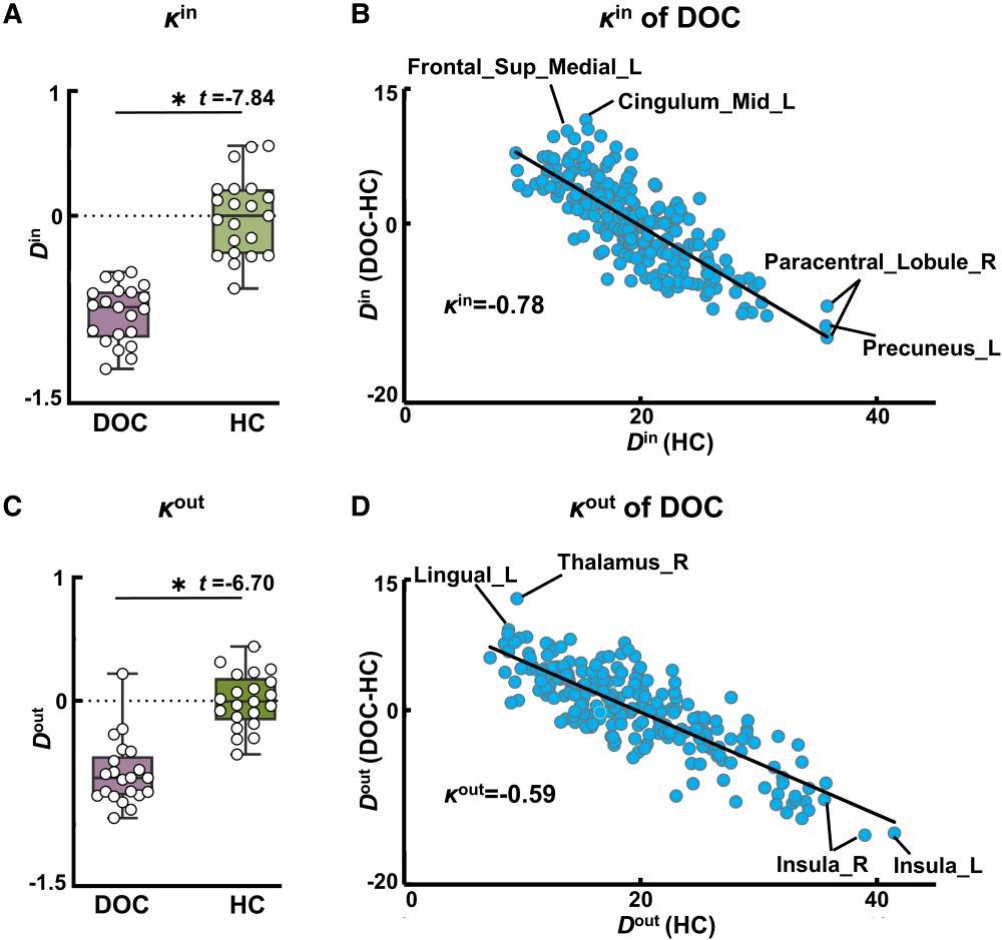
**Plots of the hub disruption index for the patients with DOC (*n* = 21) and the healthy controls (HC, *n* = 21).** (**A**) Hub disruption index of in-degree (*κ*^in^). The asterisk indicates a significant difference between the patients and the controls (*P* < 0.05, permutation test). (**B**) *κ*^in^ of the DOC patients. The *x*-axis corresponds to the mean in-degree (*D*^in^) of the controls, and the *y-*axis corresponds to the difference of the mean *D*^in^ of the patients minus the controls at each node. The slope of the fitted straight line represents the mean *κ*^in^ for the patients. The labelled nodes show the radically altered *D*^in^ in the patients compared with the controls. (**C**) Hub disruption index of out-degree (κ^out^). (**D**) *D*^out^ of the DOC patients. Sup, superior; Mid, middle; L (R), left (right) hemisphere.

Figure 3A and C shows that the *κ*^in^ and *κ*^out^ were significantly lower in the patients than in the controls. We found *D*^in^ (*D*^out^) in several nodes showing radical reorganization in the patients compared with the controls (Fig. 3B and D). Specifically, several driven hub nodes in the controls, such as the left precuneus and the right paracentral lobule, showed radically decreased *D*^in^ in the patients, whereas several driven non-hub nodes in the controls, such as the left middle cingulate gyrus and the left medial superior frontal gyrus, showed radically increased *D*^in^ in the patients. Similarly, several driving hub nodes in the controls, such as the bilateral insula, showed sharply decreased *D*^out^ in the patients, whereas several driving non-hub nodes in the controls, such as the right thalamus and the left lingual gyrus, showed radically increased *D*^out^ in the patients.

### Motif configuration analysis

**Figure 4:**
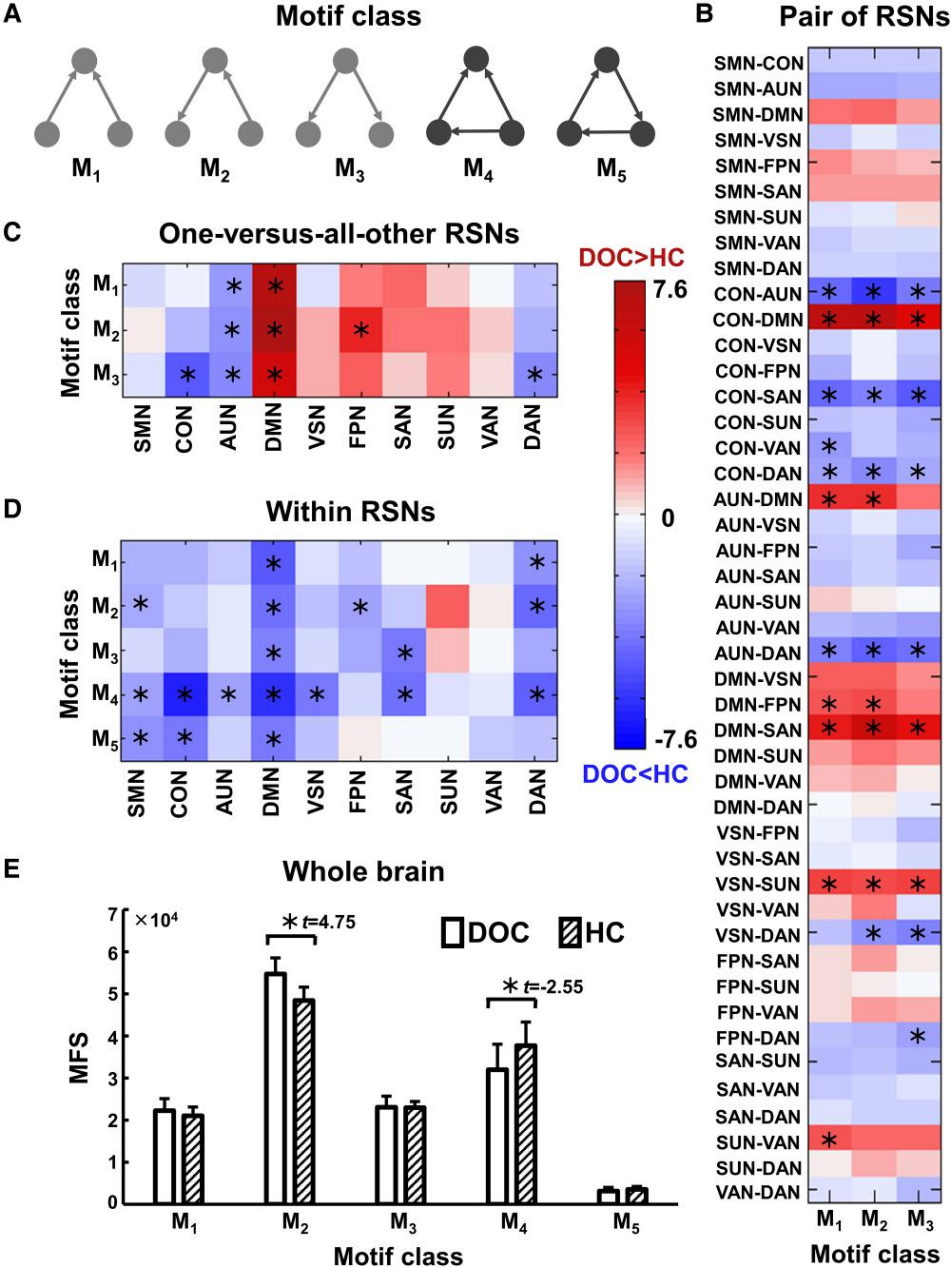
**Comparison of the MFS between the patients with DOC (*n* = 21) and the healthy controls (HC, *n* = 21).** (**A**) The five unidirectional motif classes (M_1_, M_2_, M_3_, M_4_ and M_5_) consisting of three nodes. The classes with the grey colour (M_1_, M_2_ and M_3_) are the classes of open-triangle motif, and the classes with black colour (M_4_ and M_5_) are the classes of closed-triangle motif. The *t*-value matrix of the MFS for (**B**) between pairs of RSNs, (**C**) between one-versus-all-other RSNs and (**D**) within RSNs. An asterisk indicates a significant between-group difference (*P* < 0.05, permutation test, FDR-corrected). (**E**) The mean MFS for the five motif classes in the whole brain. SMN, sensorimotor network; CON, cingulo-opercular network; AUN, auditory network; DMN, default mode network; VSN, visual network; FPN, frontoparietal network; SAN, salience network; SUN, subcortical network; VAN, ventral attention network; DAN, dorsal attention network.

Figure 4 shows the between-group differences in the MFS of five motif classes (Fig. 4A) between pairs of RSNs, between one-versus-all-other RSNs, within RSNs and in the whole brain.

*Between pairs of RSNs*: We found that only three classes of open-triangle motif (M_1_, M_2_ and M_3_) existed between the RSNs in the patients and the controls. Figure 4B shows the significant between-group differences in the MFS of the three classes between all pairs of RSNs. The patients had significantly higher MFS in all the three classes between the CON–DMN, DMN–SAN and VSN–SUN, and significantly lower MFS in all the three classes between the CON–AUN, CON–SAN, CON–DAN and AUN–DAN than the controls. The detailed information of MFS between pairs of RSNs is listed in Supplementary Table 1.

*Between one-versus-all-other RSNs*: Fig. 4C shows the significantly different MFS in three classes of open-triangle motif (M_1_, M_2_ and M_3_) between one-versus-all-other RSNs between the patients and the controls. The patients had significantly higher MFS in all the three classes between the DMN-versus-all-other RSNs and significantly lower MFS in all the three classes between the AUN-versus-all-other RSNs than the controls. The detailed information of MFS between one-versus-all-other RSNs is listed in Supplementary Table 2.

*Within RSNs*: We found that five motif classes (M_1_, M_2_, M_3_, M_4_ and M_5_) occurred within each RSN for the patients and the controls. Figure 4D shows that the patients had significantly lower MFS in the five classes within several RSNs than the controls. Particularly, the patients showed significantly lower MFS in all the five classes in the DMN than the controls. The detailed information of MFS within RSNs is listed in Supplementary Table 3.

*Whole brain*: The patients showed significantly higher MFS of class M_2_ and significantly lower MFS of class M_4_ in the whole brain compared with the controls (Fig. 4E).

### Global topology analysis

No significant difference was observed in the global efficiency between the DOC patients and the healthy controls. The global clustering coefficient was significantly lower in the patients than in the controls (*t* = −3.83, *P* < 0.001).

### Canonical correlation analysis

**Figure 5:**
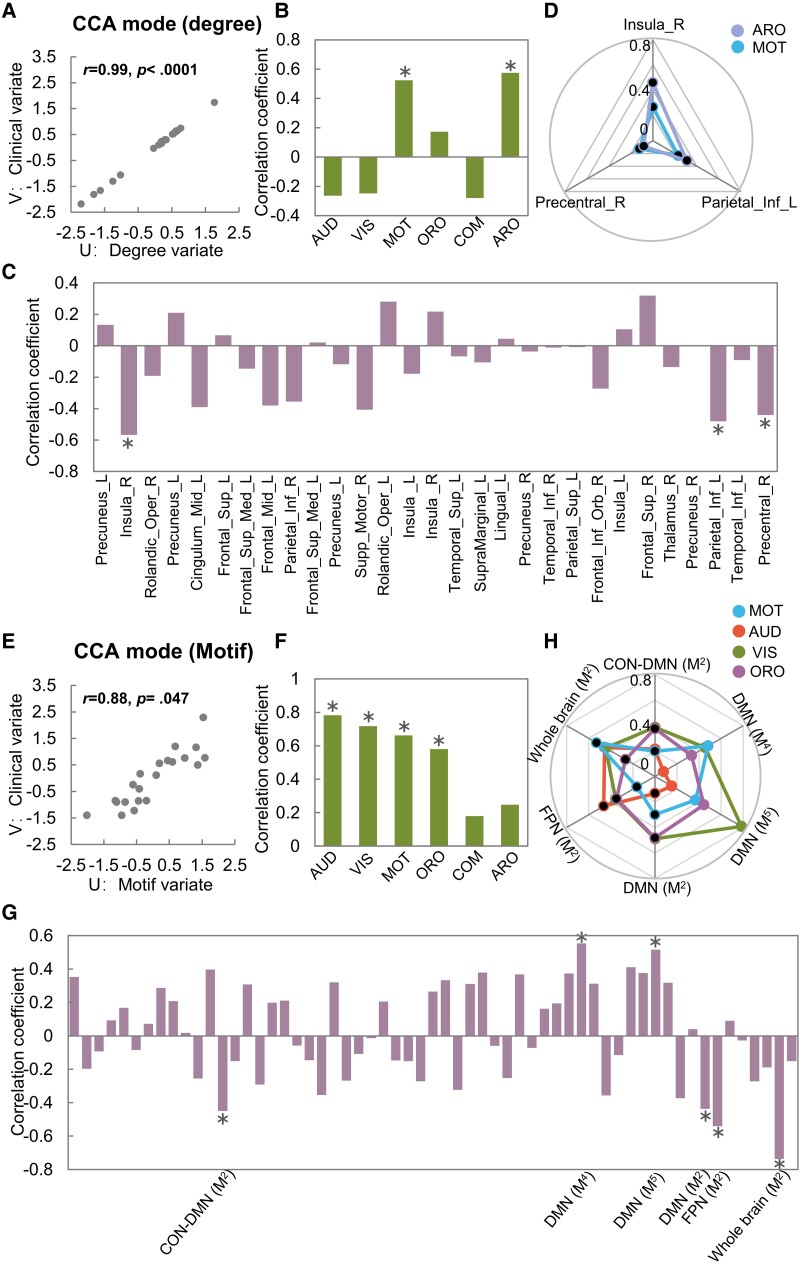
**CCA mode for degree and motif in the patients with DOC (*n* = 21).** (**A**) CCA mode for degree. (**B**) Correlation between the clinical variate of CCA mode for degree and six sub-scores of CRS-R. An asterisk indicates a significant correlation (*P* < 0.05, permutation test). (**C**) Correlation between the degree variate and original degree metrics with significant between-group differences. (**D**) Plots showing the correlation between three original degree metrics that were significantly correlated to the degree variate and two sub-scores of CRS-R that were significantly correlated to the clinical variate. The black dots represent the negative correlation coefficients. (**E**) CCA mode for motif. (**F**) Correlation between the clinical variate of CCA mode for motif and six sub-score of CRS-R. (**G**) Correlation between the motif variate and original MFS metrics with significant between-group differences. (**H**) Plots showing the correlation between six original MFS metrics that were significantly correlated to the motif variate and four sub-scores of CRS-R that were significantly correlated to the clinical variate. AUD, auditory; VIS, visual; MOT, motor; ORO, oromotor; COM, communication; ARO, arousal; Sup, superior; Inf, inferior; Mid, middle; Oper, operculum; Supp, supplementary; Orb, orbital; L (R), left (right) hemisphere; CON, cingulo-opercular network; DMN, default mode network; FPN, frontoparietal network.

Figure 5A and E shows that the degree variate and the clinical variate, as well as the motif variate and the clinical variate, were significantly correlated, respectively. No significant correlation was observed between the global variate and the clinical variate.

For CCA mode between the degree variate and the clinical variate, Fig. 5B shows that the clinical variate is significantly positively correlated with two sub-scores of CRS-R, including (i) motor and (ii) arousal. Figure 5C shows that the degree variate is significantly negatively correlated with the degree of three regions: (i) *D*^in^ of the right insula, (ii) *D*^out^ of the left inferior parietal gyrus and (iii) *D*^out^ of the right precentral gyrus. Figure 5D shows that the arousal sub-score is significantly negatively correlated with *D*^in^ of the right insula. The detailed information is listed in Supplementary Table 4.

For CCA mode between the motif variate and the clinical variate, Fig. 5F shows that the clinical variate is significantly positively correlated with four sub-scores of CRS-R, including (i) auditory, (ii) visual, (iii) motor and (iv) oromotor. Figure 5G shows that the motif variate is significantly positively correlated with the MFS of (i) M_4_ within the DMN, (ii) M_5_ within the DMN, and significantly negatively correlated with the MFS of (i) M_2_ between the CON–DMN, (ii) M_2_ between the DMN-versus-all-others RSNs, (iii) M_2_ between the FPN-versus-all-others RSNs and (iv) M_2_ within the whole brain. Figure 5H shows that the auditory sub-score is significantly negatively correlated with the MFS of (i) M_2_ between the FPN-versus-all-other RSNs and (ii) M_2_ within the whole brain. The visual sub-score is significantly positively correlated with the MFS of (i) M_4_ within the DMN and (ii) M_5_ within the DMN, and significantly negatively correlated with the MFS of (i) M_2_ between the DMN-versus-all-other RSNs and (ii) M_2_ within the whole brain. The motor sub-score is significantly positively correlated with the MFS of M_4_ within the DMN, and significantly negatively correlated with the MFS of M_2_ within the whole brain. The oromotor sub-score is significantly positively correlated with the MFS of M_5_ within the DMN and significantly negatively correlated with the MFS of M_2_ between the DMN-versus-all-other RSNs. The detailed information is listed in Supplementary Table 5.

### Robustness analysis

First, the main results were repeatable when using the non-smoothed data, which are shown in Supplementary Figs 1–3 and Supplementary Table 6. Second, when using maximum lagged correlation to construct the directed brain network, we found that the results were similar to those reported in the main text (Supplementary Figs 4–6 and Supplementary Table 6).

## Discussion

The current study constructed whole-brain directed functional networks by combining FC analysis with TD estimation, calculated multi-scale graph theoretical metrics based on the directed brain networks, and estimated relationships between the abnormal graph theoretical metrics and the sub-scores of CRS-R for DOC patients. We found disrupted directed connection patterns at multiple topological scales in DOC patients, and significant correlations between the disrupted directed connection patterns at both nodal and RSN scales with clinical scores in DOC patients.

### Disruption in nodal degree

The current study detected nodes with abnormal *D*^in^ and *D*^out^ in the DOC patients compared with the healthy controls (Fig. 2 and Table 3). Among these nodes, the precuneus, which was considered to engage in self-consciousness,^57^ showed significantly lower *D*^in^ and significantly higher *D*^out^ for the patients than the controls. Previous studies reported reduced functional connections in the precuneus in DOC patients.^11,58^ The current results further provided neural evidence about the relationships between the direction of functional connections in the precuneus and the maintenance of consciousness.

Moreover, we observed radical reorganization ranging from hub nodes to non-hub nodes in the DOC patients relative to the healthy controls (Fig. 3). The left precuneus, which was a driven hub node in the controls, became a non-hub node in the patients. This is supported by a previous study that found the precuneus was a hub node associated with the recovery of consciousness.^9^ Seguin *et al*.^59^ also suggested that the precuneus was a driven hub node in the brain networks according to the direction of information flow. Our results indicated that the capacity of the precuneus to receive information was disrupted in DOC patients, which might be due to the reduction of physical resources used to sustain the highly dense connections of hub nodes after brain damage.^60,61^

In addition, the right thalamus, which was a non-hub node in the controls, had significantly higher *D*^out^ in the patients than in the controls. Previous studies have widely reported that the loss of consciousness is linked to the disruption of thalamocortical brain connections.^17,20,62‐64^ Preller *et al*.^65^ found that increased FC between the thalamus and sensory-somatomotor region was associated with altered states of consciousness. Our results showed the emergence of radically increased connections in the right thalamus, which might mean an immediate physical response of non-hub nodes to brain damage.^7^

### Reorganization of network motif configuration

The MFS was used to detect the network motif configuration. We found significantly altered MFS in the classes of open-triangle motif (M_1_, M_2_ and M_3_) between the RSNs in the DOC patients compared with the healthy controls (Fig. 4 and Supplementary Table 1). These results are consistent with a previous electroencephalography study, which reported significantly decreased MFS of the classes of open-triangle motif in healthy subjects under anaesthesia compared with those under wakefulness.^66^ Thus, both anaesthesia-induced and injury-induced unconsciousness are associated with the topological reorganization of the open-triangle motif between RSNs. In the open-triangle motif, the node connecting to the other two nodes is a relay station for information communication and plays a crucial role in the integration of segregated networks.^52,67^ The topological reorganization of the open-triangle motif may imply disrupted functional integration between RSNs in the DOC patients.

Furthermore, we observed that the DOC patients showed significantly lower MFS in the classes of closed-triangle motif (M_4_ and M_5_) within RSNs than in the healthy controls (Fig. 4 and Supplementary Table 3). In the closed-triangle motif, the inter-nodal information communication does not necessarily go through a third node. The loop-pattern communication may greatly contribute to sustaining local clustering,^50^ and the classes of closed-triangle motif have been detected within RSNs.^52^ Thus, our results of a lower MFS in the classes of closed-triangle motif may indicate a defect in local segregation in the DOC patients. Taken together, the altered MFS in the open-triangle motif between RSNs and the closed-triangle motif within RSNs in the DOC patients may reflect the abnormal information flow underlying the impaired consciousness.

Notably, we found significantly higher MFS in all three classes of open-triangle motif (M_1_, M_2_ and M_3_) between the DMN-versus-all-other RSNs in the patients than in the controls (Fig. 4 and Supplementary Table 2). These results are supported by previous studies, which found abnormal FC between the DMN and other RSNs in DOC patients.^11,68‐70^ Moreover, we observed that the patients showed significantly lower MFS in all the five motif classes within the DMN than the controls (Fig. 4 and Supplementary Table 3). Previous studies have found reduced white matter fibre connections ^10^ and functional connections ^71^ between several regions within the DMN in DOC patients. A recent case study^72^ showed that motif configurations within the DMN can predict the clinical recovery of DOC patients. We inferred that the three-node connection patterns within and between the DMN might be essential to the maintenance of conscious activity.

### Alteration of global topological properties

No significant difference was observed in the global efficiency between the DOC patients and the healthy controls, which was consistent with several previous rs-fMRI studies.^7,8,73^ However, we found a significantly lower global clustering coefficient in the patients than in the controls, which was in line with previous studies,^69,74^ suggesting deficient functional segregation and low-efficiency information transmission in the brain networks at the global scale for DOC patients. This result may be associated with the significantly reduced MFS of the classes of closed-triangle motif within RSNs for the DOC patients, because the closed-triangle motif was thought to play a pivotal role in local clustering and functional segregation of the brain networks.^50,52^ Future studies could focus on the relationships between motif within or between RSNs and brain segregation or integration under different states of consciousness.

We also noticed abnormal connection patterns in the DMN across multiple topological scales for the DOC patients. At the nodal scale, we observed that the precuneus, a strong driven hub of the DMN,^59,75^ showed a reduced capacity of information receiving in the patients. At the RSN scale, we found a reorganized three-node motif pattern within and between the DMN in the patients. Taken together, we inferred that the connections centred on the precuneus in the DMN might be extensively impaired in DOC patients. Furthermore, the disrupted directed connection patterns at the nodal scale and the RSN scale may lead to the alteration of the global topological organization for DOC patients.

### Correlations between graph theoretical metrics and clinical scores

In this study, CCA was applied to detect the correlations between abnormal graph theoretical metrics at each topological scale and clinical scores in DOC patients. We found a significant correlation between the abnormal degree metrics and the sub-scores of CRS-R (Fig. 5), suggesting that the directed connection patterns between brain regions were related to the clinical performances in DOC patients. Notably, we observed a significant correlation between the *D*^in^ of the right insula and the arousal sub-score of CRS-R in the patients. The arousal sub-score indicates the level of wakefulness of DOC patients. Fischer *et al*.^76^ used the lesion network mapping approach to characterize the functional brain network localized to coma-causing brainstem lesions and found that the FC between the brainstem and the anterior insula was crucial to the maintenance of arousal. In fact, as a hub region, the insula contributes to receiving different sensory information and has been considered as an important component of conscious processing.^77,78^ Our results complemented new evidence on the importance of steady information flow in the insula for the maintenance of arousal.

We also observed a significant correlation between the abnormal MFS metrics and the sub-scores of CRS-R in the DOC patients (Fig. 5). This result indicated that the altered three-node topological configurations in directed brain networks might be bound up with impaired conscious activity. Moreover, the results of Pearson’s correlation analysis suggested that the motif configurations involving the DMN were correlated with several sub-scores of CRS-R, including auditory, visual, motor and oromotor. This may indicate that the information flow in the DMN is sensitive to the behavioural responses of DOC patients. Wu *et al*.^79^ also found that the local FC strength and fractional amplitude of low-frequency fluctuations in the DMN were significantly positively correlated with the oromotor and motor sub-scores of CRS-R. In addition, taking the inferior parietal lobe (located in DMN) as a target, the transcranial direct current stimulation and the repetitive transcranial magnetic stimulation can prominently improve the clinical performances of MCS patients.^80^ Thus, we inferred that the DMN plays a pivotal role in maintaining awareness of perceptible surroundings.^10,12^

### Limitations

Several limitations should be addressed. First, the current study analysed the rs-fMRI data from 21 DOC patients with different states of consciousness (VS and MCS), which may affect the generalizability of the results. Further studies need to collect a large sample and classify DOC patients into distinct sub-types for detecting the directed brain network organization related to impaired consciousness. Second, the selection of the brain template may affect the results. In the current study, we selected the Power-264 template^32^ to construct the directed brain networks because of its reasonable partitioning and extensive use.^8,13,35‐39^ However, there was no established standard for assessing template suitability when constructing brain networks. In addition, we regressed out the global signal when preprocessing the rs-fMRI data to perform TDE.^21^ Although a portion of the global signal may include neural activity,^81,82^ the majority of the components of the global signal contain artefacts related to physiological noises and head motions.^83‐85^ Because there was no standard method to extract the true signal and denoise the artefacts, the analysis without global signal regression is likely to bias the results of network estimation.^86^

## Conclusion

The current study analysed the brain network organization at multiple topological scales based on directed functional connections for DOC patients. At the nodal scale, the patients showed abnormal directed connection patterns in the precuneus and the thalamus. At the RSN scale, we observed the reorganization of three-node directed connection patterns within and between DMN in the patients. At the global scale, we found significantly decreased functional segregation in the patients. Furthermore, the CCA showed the clinical correlations of directed connection patterns at both nodal and RSN scales. The findings may suggest that the disruptions of whole-brain directed interactions are associated with clinical characteristics of impaired consciousness.

## Supplementary Material

fcad069_Supplementary_Data

## Data Availability

The data that support the findings of this study are available from the corresponding author upon reasonable request.

## Abbreviations

AUN =: auditory network
CCA =: canonical correlation analysis
CCF =: cross-covariance function
CON =: cingulo-opercular network
CRS-R =: Coma Recovery Scale-Revised
DAN =: dorsal attention network
DCM =: dynamic causal modelling
DMN =: default mode network
DOC =: disorders of consciousness
FA =: flip angle
FC =: functional connectivity
fMRI =: functional magnetic resonance imaging
FOV =: field of view
FPN =: frontoparietal network
MCS =: minimally conscious state
MFS =: motif frequency spectrum
ROI =: region of interest
RSN =: resting-state network
SAN =: salience network
SMN =: sensorimotor network
SUN =: subcortical network
TD =: time delay
TDE =: time delay estimation
TE =: echo time
TR =: repetition time
VAN =: ventral attention network
VS/UWS =: vegetable state/unresponsive wakefulness syndrome
VSN =: visual network
