# Supplementary material for: Disrupted multi-scale topological organization of directed functional brain networks in patients with disorders of consciousness
Source: Brain Commun. 2023 Mar 28;5(2):fcad069. doi: 10.1093/braincomms/fcad069 (PMC10066573; doi:10.1093/braincomms/fcad069)
Supplement: fcad069_Supplementary_Data [file fcad069_supplementary_data.docx]

**Supplementary Material**

**Materials and methods**

Nodal degree

The in-degree (*D*^in^) and out-degree (*D*^out^) for each node were calculated according to the definition in Rubinov and Sporns ^1^. The *D*^in^ of node *i* was obtained by counting the number of incoming edges in the *i*^th^ column in the directed functional connection matrix and is given by:

$$\begin{aligned} D_{i}^{in}=\sum_{j} A_{ij}^{in}, (1) \end{aligned}$$

where *i* and *j* represent two nodes in the brain network, and $A_{ij}^{in}$ represents the incoming edge of node *i* connecting with another node *j,* and the direction is from *j* to *i*.

The *D*^out^ of node *i* was defined by counting the number of outgoing edges in *i*^th^ raw and is given by:

$$\begin{aligned} D_{i}^{out}=\sum_{j} A_{ij}^{out}, (2) \end{aligned}$$

where $A_{ij}^{out}$ represents the outgoing edge of node *i* connecting with another node *j,* and the direction is from node *i* to *j*.

Motif frequency spectrum

The motif frequency spectrum (MFS) counts the number of occurrences of each motif class in a certain network ^2^ and is given by:

$$\begin{aligned} F_{M_{h}}^{\left( h' \right)}=\sum_{i} J_{h^{'},i}, (3) \end{aligned}$$

where $h'$ represents any motif class whose node number is *M_h_*, and $J_{h^{'},i}$ is the number of occurrences of motif class *h’* around node *i*.

Global efficiency

The global efficiency ^1,3^ measures information flow throughout the whole-brain network, which indicates the functional integration of the brain network. It was calculated by the mean of the inverse of shortest path length (a path with the minimum number of edges between two nodes) between all pairs of nodes in the brain network and is given by:

$$\begin{aligned} E^{\to}=\frac{1}{N}\sum_{i} \frac{\sum_{i\neq j} \left( d_{ij}^{\to} \right)^{-1}}{N-1}, (4) \end{aligned}$$

where *N* represents the number of total nodes in the brain network; $d_{ij}^{\to}$ represents the shortest path length from node *i* to node *j.* The global efficiency with a higher value implies stronger global integration of the brain network.

Global clustering coefficient

The clustering coefficient can be measured at the nodal and global scale. At the nodal scale, the clustering coefficient of a node represents the proportion of the definite edges to the possible edges between the node's neighbors and reflects the local efficiency of information transfer between the node's neighbors ^4,5^. The mean clustering coefficient for all nodes in the whole brain is defined as the global clustering coefficient and is given by:

$$\begin{aligned} C^{\to}=\frac{1}{N}\sum_{i} \frac{t_{i}^{\to}}{\left( K_{i}^{out}+K_{i}^{in} \right)\left( K_{i}^{out}+K_{i}^{in}-1 \right)}, (5) \end{aligned}$$

where $t_{i}^{\to}$ is the number of directed triangles around node *i.* A higher global clustering coefficient indicates stronger functional segregation of the brain network.

The *D*^in^ and *D*^out^, MFS, global efficiency, and global clustering coefficient were calculated using the Brain Connectivity Toolbox (<https://www.nitrc.org/projects/bct/>).

Canonical correlation analysis

Canonical correlation analysis (CCA) is a multivariate approach that allows detecting the association between imaging data and clinical scores. In this study, CCA mode was estimated by computing the maximal correction between the graph theoretical variate which is the linear combination of the abnormal graph theoretical metrics at any topological scale and the clinical variate which is the linear combination of 6 sub-scores of CRS-R in the DOC patients. Specifically, we first constructed graph theoretical metrices at each topological scale and clinical matrices, X^1^_n🞩s1_ and Y^1^_n🞩t_ (n = 21 DOC patients, S1 = 29 *D*^in^ and *D*^out^ metrics with significant between-group differences, *t* = 6 sub-scores of CRS-R), X^2^_n🞩s2_ and Y^2^_n🞩t_ (S2 = 59 MFS metrics with significant between-group differences) and X^3^_n🞩s3_ and Y^3^_n🞩t_ (S3 = 1 global metric with significant between-group difference). Then, each CCA mode that estimated the maximal correlation between the graph theoretical variate (U^1^ = X^1^A^1^, U^2^ = X^2^A^2^ or U^3^=X^3^A^3^) and the clinical variate (V^1^ = Y^1^B^1^, V^2^ = Y^2^B^2^ or V^3^ = Y^3^B^3^) was obtained by using *canoncorr.m* in MATLAB. Vectors A and B represent the weights to define the graph theoretical variate and the clinical variate, respectively.

**References**

1. Rubinov M, Sporns O. Complex network measures of brain connectivity: Uses and interpretations. *Neuroimage*. 2010;52(3):1059-1069. doi:10.1016/j.neuroimage.2009.10.003

2. Sporns O, Kotter R. Motifs in brain networks. *PLoS Biol*. 2004;2(11):e369. doi:10.1371/journal.pbio.0020369

3. Latora V, Marchiori M. Efficient behavior of small-world networks. *Phys Rev Lett*. 2001;87(19):198701. doi:10.1103/PhysRevLett.87.198701

4. Watts DJ, Strogatz SH. Collective Dynamics of Small World Networks. *Nature*. 1998;393(6684):440-442. doi:10.1038/30918

5. Fagiolo G. Clustering in complex directed networks. *Phys Rev E Stat Nonlin Soft Matter Phys*. 2007;76(2 Pt 2):026107. doi:10.1103/PhysRevE.76.026107

**Results**

**Supplementary Table 1** Comparison of the motif frequency spectrum (MFS) for pairs of RSNs between the patients with disorders of consciousness (DOC) and the healthy controls (HC)

| Motif class | Between RSNs | Motif frequency spectrum | | *t-*value | *p-*value |
| --- | --- | --- | --- | --- | --- |
|  |  | DOC | HC |  |  |
| M_1_ | SMN-CON | 385 | 515 | -1.46 | 1.46×10^-1^ |
|  | SMN-AUN | 364 | 679 | -2.43 | 2.08×10^-2^ |
|  | SMN-DMN | 396 | 261 | 1.99 | 4.60×10^-2^ |
|  | SMN-VSN | 219 | 488 | -1.60 | 1.25×10^-1^ |
|  | SMN-FPN | 228 | 134 | 1.55 | 1.31×10^-1^ |
|  | SMN-SAN | 179 | 137 | 1.29 | 2.13×10^-1^ |
|  | SMN-SUN | 104 | 114 | -0.82 | 4.22×10^-1^ |
|  | SMN-VAN | 62 | 111 | -1.57 | 1.27×10^-1^ |
|  | SMN-DAN | 143 | 193 | -1.40 | 1.73×10^-1^ |
|  | CON-AUN | 99 | 203 | -4.51 | 4.00×10^-4*^ |
|  | CON-DMN | 183 | 49 | 6.42 | 2.00×10^-4*^ |
|  | CON-VSN | 41 | 67 | -1.40 | 1.73×10^-1^ |
|  | CON-FPN | 77 | 113 | -2.32 | 2.54×10^-2^ |
|  | CON-SAN | 106 | 244 | -4.36 | 2.00×10^-4*^ |
|  | CON-SUN | 71 | 106 | -1.77 | 8.80×10^-2^ |
|  | CON-VAN | 29 | 70 | -3.03 | 2.60×10^-3*^ |
|  | CON-DAN | 18 | 71 | -2.65 | 6.00×10^-4*^ |
|  | AUN-DMN | 143 | 56 | 3.66 | 6.00×10^-4*^ |
|  | AUN-VSN | 53 | 97 | -1.21 | 2.58×10^-1^ |
|  | AUN-FPN | 41 | 59 | -1.61 | 1.17×10^-1^ |
|  | AUN-SAN | 46 | 72 | -1.70 | 9.26×10^-2^ |
|  | AUN-SUN | 71 | 59 | 0.62 | 5.42×10^-1^ |
|  | AUN-VAN | 35 | 49 | -1.96 | 5.82×10^-2^ |
|  | AUN-DAN | 16 | 50 | -3.59 | 2.00×10^-4*^ |
|  | DMN-VSN | 467 | 250 | 2.54 | 1.70×10^-2^ |
|  | DMN-FPN | 877 | 583 | 2.71 | 7.60×10^-3*^ |
|  | DMN-SAN | 518 | 260 | 4.15 | 8.00×10^-4*^ |
|  | DMN-SUN | 199 | 139 | 1.28 | 1.98×10^-1^ |
|  | DMN-VAN | 183 | 163 | 0.78 | 4.38×10^-1^ |
|  | DMN-DAN | 120 | 111 | -0.10 | 9.21×10^-1^ |
|  | VSN-FPN | 143 | 132 | -0.26 | 7.96×10^-1^ |
|  | VSN-SAN | 61 | 83 | -0.67 | 5.31×10^-1^ |
|  | VSN-SUN | 68 | 13 | 3.22 | 4.00×10^-4*^ |
|  | VSN-VAN | 55 | 32 | 0.55 | 5.76×10^-1^ |
|  | VSN-DAN | 130 | 186 | -1.69 | 9.12×10^-2^ |
|  | FPN-SAN | 265 | 237 | 0.41 | 6.97×10^-1^ |
|  | FPN-SUN | 43 | 37 | 0.28 | 7.76×10^-1^ |
|  | FPN-VAN | 45 | 51 | 0.35 | 7.34×10^-1^ |
|  | FPN-DAN | 82 | 108 | -1.73 | 8.26×10^-2^ |
|  | SAN-SUN | 51 | 75 | -1.90 | 6.42×10^-2^ |
|  | SAN-VAN | 31 | 59 | -1.60 | 1.25×10^-1^ |
|  | SAN-DAN | 21 | 36 | -0.77 | 6.01×10^-1^ |
|  | SUN-VAN | 32 | 14 | 2.66 | 9.20×10^-3*^ |
|  | SUN-DAN | 9 | 12 | 0.18 | 8.63×10^-1^ |
|  | VAN-DAN | 9 | 13 | -0.94 | 3.64×10^-1^ |
| M_2_ | SMN-CON | 554 | 727 | -1.52 | 1.41×10^-1^ |
|  | SMN-AUN | 573 | 1,018 | -2.50 | 1.82×10^-2^ |
|  | SMN-DMN | 639 | 388 | 2.36 | 2.48×10^-2^ |
|  | SMN-VSN | 326 | 497 | -0.65 | 5.85×10^-1^ |
|  | SMN-FPN | 317 | 215 | 1.05 | 3.01×10^-1^ |
|  | SMN-SAN | 290 | 218 | 1.41 | 1.76×10^-1^ |
|  | SMN-SUN | 152 | 166 | -0.62 | 5.34×10^-1^ |
|  | SMN-VAN | 127 | 188 | -0.96 | 3.43×10^-1^ |
|  | SMN-DAN | 227 | 319 | -1.31 | 2.07×10^-1^ |
|  | CON-AUN | 123 | 277 | -5.89 | 2.00×10^-4*^ |
|  | CON-DMN | 307 | 67 | 6.83 | 2.00×10^-4*^ |
|  | CON-VSN | 69 | 77 | -0.29 | 7.73×10^-1^ |
|  | CON-FPN | 118 | 120 | -0.41 | 7.12×10^-1^ |
|  | CON-SAN | 176 | 326 | -3.61 | 1.40×10^-3*^ |
|  | CON-SUN | 98 | 139 | -1.48 | 1.52×10^-1^ |
|  | CON-VAN | 49 | 72 | -1.49 | 1.47×10^-1^ |
|  | CON-DAN | 30 | 66 | -3.56 | 1.40×10^-3*^ |
|  | AUN-DMN | 240 | 84 | 3.76 | 4.00×10^-4*^ |
|  | AUN-VSN | 84 | 119 | -0.64 | 5.34×10^-1^ |
|  | AUN-FPN | 54 | 76 | -1.33 | 1.94×10^-1^ |
|  | AUN-SAN | 67 | 94 | -1.42 | 1.61×10^-1^ |
|  | AUN-SUN | 87 | 76 | 0.15 | 8.82×10^-1^ |
|  | AUN-VAN | 53 | 72 | -2.36 | 2.58×10^-2^ |
|  | AUN-DAN | 20 | 64 | -4.62 | 2.00×10^-4*^ |
|  | DMN-VSN | 730 | 392 | 2.55 | 1.36×10^-2^ |
|  | DMN-FPN | 1,386 | 896 | 2.92 | 5.00×10^-3*^ |
|  | DMN-SAN | 888 | 379 | 5.81 | 2.00×10^-4*^ |
|  | DMN-SUN | 321 | 184 | 1.94 | 5.54×10^-2^ |
|  | DMN-VAN | 314 | 264 | 1.15 | 2.61×10^-1^ |
|  | DMN-DAN | 190 | 183 | 0.08 | 9.34×10^-1^ |
|  | VSN-FPN | 195 | 195 | -0.83 | 4.06×10^-1^ |
|  | VSN-SAN | 85 | 110 | -0.44 | 6.98×10^-1^ |
|  | VSN-SUN | 92 | 18 | 2.95 | 3.40×10^-3*^ |
|  | VSN-VAN | 59 | 30 | 1.77 | 7.94×10^-2^ |
|  | VSN-DAN | 165 | 238 | -3.10 | 5.20×10^-3*^ |
|  | FPN-SAN | 394 | 320 | 1.30 | 2.13×10^-1^ |
|  | FPN-SUN | 44 | 43 | 0.08 | 9.39×10^-1^ |
|  | FPN-VAN | 79 | 73 | 1.27 | 2.09×10^-1^ |
|  | FPN-DAN | 113 | 145 | -2.10 | 3.86×10^-2^ |
|  | SAN-SUN | 78 | 108 | -1.83 | 7.10×10^-2^ |
|  | SAN-VAN | 48 | 78 | -1.26 | 2.17×10^-1^ |
|  | SAN-DAN | 29 | 42 | -1.21 | 2.35×10^-1^ |
|  | SUN-VAN | 41 | 19 | 2.32 | 2.42×10^-2^ |
|  | SUN-DAN | 14 | 13 | 1.00 | 3.14×10^-1^ |
|  | VAN-DAN | 13 | 16 | -0.62 | 5.44×10^-1^ |
| M_3_ | SMN-CON | 411 | 584 | -1.53 | 1.35×10^-1^ |
|  | SMN-AUN | 463 | 803 | -2.22 | 2.88×10^-2^ |
|  | SMN-DMN | 401 | 323 | 1.29 | 2.03×10^-1^ |
|  | SMN-VSN | 223 | 456 | -1.35 | 2.01×10^-1^ |
|  | SMN-FPN | 245 | 174 | 0.87 | 3.98×10^-1^ |
|  | SMN-SAN | 226 | 163 | 1.34 | 1.99×10^-1^ |
|  | SMN-SUN | 142 | 141 | 0.39 | 7.10×10^-1^ |
|  | SMN-VAN | 120 | 178 | -1.02 | 3.24×10^-1^ |
|  | SMN-DAN | 167 | 283 | -1.62 | 9.40×10^-2^ |
|  | CON-AUN | 105 | 211 | -3.96 | 1.40×10^-3*^ |
|  | CON-DMN | 198 | 61 | 4.90 | 2.00×10^-4*^ |
|  | CON-VSN | 48 | 98 | -1.58 | 1.20×10^-1^ |
|  | CON-FPN | 85 | 119 | -1.80 | 7.88×10^-2^ |
|  | CON-SAN | 113 | 272 | -4.84 | 2.00×10^-4*^ |
|  | CON-SUN | 74 | 127 | -2.42 | 1.94×10^-2^ |
|  | CON-VAN | 31 | 52 | -2.25 | 3.22×10^-2^ |
|  | CON-DAN | 23 | 62 | -2.51 | 5.40×10^-3*^ |
|  | AUN-DMN | 149 | 76 | 2.07 | 4.28×10^-2^ |
|  | AUN-VSN | 60 | 134 | -1.62 | 1.17×10^-1^ |
|  | AUN-FPN | 39 | 74 | -2.43 | 1.90×10^-2^ |
|  | AUN-SAN | 48 | 80 | -1.79 | 7.36×10^-2^ |
|  | AUN-SUN | 76 | 75 | -0.14 | 8.84×10^-1^ |
|  | AUN-VAN | 37 | 60 | -2.68 | 1.46×10^-2^ |
|  | AUN-DAN | 15 | 56 | -4.18 | 2.00×10^-4*^ |
|  | DMN-VSN | 492 | 333 | 1.48 | 1.45×10^-1^ |
|  | DMN-FPN | 900 | 676 | 1.75 | 8.82×10^-2^ |
|  | DMN-SAN | 569 | 272 | 4.51 | 2.00×10^-4*^ |
|  | DMN-SUN | 216 | 144 | 1.43 | 1.74×10^-1^ |
|  | DMN-VAN | 210 | 217 | 0.06 | 9.59×10^-1^ |
|  | DMN-DAN | 130 | 157 | -0.63 | 5.21×10^-1^ |
|  | VSN-FPN | 128 | 180 | -2.05 | 4.46×10^-2^ |
|  | VSN-SAN | 72 | 115 | -1.03 | 3.47×10^-1^ |
|  | VSN-SUN | 66 | 17 | 3.20 | 1.20×10^-3*^ |
|  | VSN-VAN | 33 | 42 | -0.79 | 5.03×10^-1^ |
|  | VSN-DAN | 98 | 283 | -3.75 | 2.00×10^-4*^ |
|  | FPN-SAN | 260 | 261 | 0.02 | 9.81×10^-1^ |
|  | FPN-SUN | 41 | 38 | -0.04 | 9.67×10^-1^ |
|  | FPN-VAN | 57 | 52 | 1.18 | 2.59×10^-1^ |
|  | FPN-DAN | 72 | 108 | -2.76 | 7.80×10^-3*^ |
|  | SAN-SUN | 53 | 85 | -2.19 | 3.14×10^-2^ |
|  | SAN-VAN | 31 | 47 | -0.94 | 3.70×10^-1^ |
|  | SAN-DAN | 20 | 35 | -1.25 | 2.23×10^-1^ |
|  | SUN-VAN | 31 | 16 | 2.16 | 3.78×10^-2^ |
|  | SUN-DAN | 12 | 12 | 0.64 | 5.42×10^-1^ |
|  | VAN-DAN | 8 | 14 | -2.03 | 5.00×10^-2^ |

*Note.* ^*^ *p* < .05 (permutation test, FDR-corrected). A positive (negative) *t*-value indicates that the MFS between a pair of RSNs in the patients was higher (lower) than in the controls.

Abbreviations: RSN, resting-state network; SMN, sensorimotor network; CON, cingulo-opercular network; AUN, auditory network; DMN, default mode network; VSN, visual network; FPN, fronto-parietal network; SAN, salience network; SUN, subcortical network; VAN, ventral attention network; DAN, dorsal attention network

**Supplementary Table 2** Comparison of the motif frequency spectrum (MFS) for one-versus-all-others RSNs between the patients with disorders of consciousness (DOC) and the healthy controls (HC)

| Motif class | RSN | Motif frequency spectrum | | *t-*value | *p-*value |
| --- | --- | --- | --- | --- | --- |
|  |  | DOC | HC |  |  |
| M_1_ | SMN | 6,062 | 6,952 | -0.97 | 3.43×10^-1^ |
|  | CON | 3,792 | 3,923 | -0.33 | 7.65×10^-1^ |
|  | AUN | 2,948 | 4,226 | -3.01 | 3.20×10^-3*^ |
|  | DMN | 7,387 | 3,749 | 7.07 | 2.00×10^-4*^ |
|  | VSN | 3,256 | 3,782 | -0.81 | 4.47×10^-1^ |
|  | FPN | 5,091 | 4,023 | 1.76 | 9.20×10^-2^ |
|  | SAN | 4,194 | 3,536 | 2.17 | 4.04×10^-2^ |
|  | SUN | 2,195 | 1,819 | 0.54 | 5.85×10^-1^ |
|  | VAN | 2,085 | 2,194 | -0.21 | 8.46×10^-1^ |
|  | DAN | 2,105 | 2,712 | -1.69 | 1.05×10^-1^ |
| M_2_ | SMN | 9,671 | 9,861 | 0.02 | 9.87×10^-1^ |
|  | CON | 6,713 | 7,976 | -2.00 | 5.90×10^-2^ |
|  | AUN | 5,707 | 7,342 | -3.20 | 4.40×10^-3*^ |
|  | DMN | 12,520 | 6,153 | 7.54 | 2.00×10^-4*^ |
|  | VSN | 5,654 | 4,615 | 1.04 | 3.02×10^-1^ |
|  | FPN | 8,529 | 6,310 | 3.84 | 4.00×10^-4*^ |
|  | SAN | 7,504 | 6,363 | 2.06 | 4.20×10^-2^ |
|  | SUN | 4,268 | 3,022 | 1.93 | 6.18×10^-2^ |
|  | VAN | 3,930 | 3,906 | 0.49 | 6.43×10^-1^ |
|  | DAN | 3,558 | 4,627 | -2.36 | 2.20×10^-2^ |
| M_3_ | SMN | 5,558 | 6,332 | -0.87 | 4.03×10^-1^ |
|  | CON | 3,973 | 6,389 | -4.85 | 2.00×10^-4*^ |
|  | AUN | 3,619 | 4,939 | -3.51 | 1.20×10^-3*^ |
|  | DMN | 7,158 | 4,294 | 5.61 | 2.00×10^-4*^ |
|  | VSN | 3,539 | 2,925 | 1.06 | 3.06×10^-1^ |
|  | FPN | 4,857 | 3,933 | 2.45 | 1.78×10^-2^ |
|  | SAN | 4,454 | 4,245 | 0.55 | 5.93×10^-1^ |
|  | SUN | 2,960 | 2,312 | 1.76 | 8.02×10^-2^ |
|  | VAN | 2,449 | 2,515 | 0.36 | 7.38×10^-1^ |
|  | DAN | 2,027 | 3,131 | -3.47 | 6.00×10^-4*^ |

*Note.* ^*^ *p* < .05 (permutation test, FDR-corrected). A positive (negative) *t*-value indicates that the MFS for the one-versus-all-others RSN in the patients was higher (lower) than in the controls.

Abbreviations: RSN, resting-state network; SMN, sensorimotor network; CON, cingulo-opercular network; AUN, auditory network; DMN, default mode network; VSN, visual network; FPN, fronto-parietal network; SAN, salience network; SUN, subcortical network; VAN, ventral attention network; DAN, dorsal attention network

**Supplementary Table 3** Comparison of motif frequency spectrum (MFS) within RSNs between the patients with disorders of consciousness (DOC) and the healthy controls (HC)

| Motif class | RSN | Motif frequency spectrum | | *t-*value | *p-*value |
| --- | --- | --- | --- | --- | --- |
|  |  | DOC | HC |  |  |
| M_1_ | SMN | 273 | 346 | -2.19 | 3.74×10^-2^ |
|  | CON | 15 | 22 | -2.18 | 3.54×10^-2^ |
|  | AUN | 14 | 19 | -1.53 | 1.28×10^-1^ |
|  | DMN | 604 | 1,125 | -4.88 | 2.00×10^-4*^ |
|  | VSN | 197 | 214 | -0.90 | 3.72×10^-1^ |
|  | FPN | 71 | 86 | -1.83 | 7.72×10^-2^ |
|  | SAN | 32 | 29 | -0.05 | 9.56×10^-1^ |
|  | SUN | 15 | 15 | -0.07 | 9.56×10^-1^ |
|  | VAN | 3 | 3 | -0.50 | 6.38×10^-1^ |
|  | DAN | 4 | 6 | -3.32 | 2.20×10^-3*^ |
| M_2_ | SMN | 660 | 773 | -2.61 | 1.32×10^-2*^ |
|  | CON | 34 | 44 | -1.56 | 1.30×10^-1^ |
|  | AUN | 31 | 35 | -0.60 | 5.43×10^-1^ |
|  | DMN | 1,409 | 2,161 | -4.12 | 2.00×10^-4*^ |
|  | VSN | 425 | 498 | -1.75 | 9.60×10^-2^ |
|  | FPN | 171 | 217 | -2.69 | 1.00×10^-2*^ |
|  | SAN | 72 | 79 | -1.56 | 1.30×10^-1^ |
|  | SUN | 42 | 34 | 2.38 | 2.36×10^-2^ |
|  | VAN | 6 | 7 | 0.13 | 8.99×10^-1^ |
|  | DAN | 9 | 17 | -4.48 | 4.00×10^-4*^ |
| M_3_ | SMN | 270 | 295 | -1.10 | 2.90×10^-1^ |
|  | CON | 13 | 23 | -2.36 | 2.08×10^-2^ |
|  | AUN | 14 | 16 | -0.70 | 4.85×10^-1^ |
|  | DMN | 620 | 972 | -3.77 | 6.00×10^-4*^ |
|  | VSN | 196 | 223 | -1.09 | 2.84×10^-1^ |
|  | FPN | 82 | 110 | -2.54 | 1.44×10^-2^ |
|  | SAN | 28 | 46 | -3.93 | 1.00×10^-3*^ |
|  | SUN | 17 | 15 | 0.91 | 3.55×10^-1^ |
|  | VAN | 3 | 3 | -0.10 | 9.24×10^-1^ |
|  | DAN | 4 | 7 | -2.40 | 2.06×10^-2^ |
| M_4_ | SMN | 926 | 1,610 | -2.62 | 1.04×10^-2*^ |
|  | CON | 43 | 144 | -6.60 | 2.00×10^-4*^ |
|  | AUN | 55 | 95 | -2.82 | 7.20×10^-3*^ |
|  | DMN | 1,049 | 2,859 | -6.00 | 2.00×10^-4*^ |
|  | VSN | 552 | 1,169 | -3.90 | 8.00×10^-4*^ |
|  | FPN | 233 | 310 | -1.03 | 3.26×10^-1^ |
|  | SAN | 70 | 141 | -4.17 | 4.00×10^-4*^ |
|  | SUN | 87 | 102 | -0.90 | 3.78×10^-1^ |
|  | VAN | 6 | 8 | -0.70 | 4.89×10^-1^ |
|  | DAN | 6 | 25 | -4.66 | 2.00×10^-4*^ |
| M_5_ | SMN | 58 | 111 | -3.20 | 3.20×10^-3*^ |
|  | CON | 3 | 13 | -4.01 | 4.00×10^-4*^ |
|  | AUN | 5 | 7 | -1.33 | 1.90×10^-1^ |
|  | DMN | 96 | 234 | -3.85 | 6.00×10^-4*^ |
|  | VSN | 46 | 60 | -1.72 | 9.80×10^-2^ |
|  | FPN | 23 | 21 | 0.18 | 8.69×10^-1^ |
|  | SAN | 7 | 7 | -0.16 | 8.78×10^-1^ |
|  | SUN | 6 | 7 | -0.21 | 8.33×10^-1^ |
|  | VAN | 1 | 1 | -1.56 | 1.28×10^-1^ |
|  | DAN | 1 | 2 | -1.72 | 5.02×10^-2^ |

*Note*. ^*^ *p* < .05 (permutation test, FDR-corrected). A positive (negative) *t*-value indicates that the MFS within the RSN was higher (lower) in the patients than in the controls.

Abbreviations: RSN, resting-state network; SMN, sensorimotor network; CON, cingulo-opercular network; AUN, auditory network; DMN, default mode network; VSN, visual network; FPN, fronto-parietal network; SAN, salience network; SUN, subcortical network; VAN, ventral attention network; DAN, dorsal attention network

**Supplementary Table 4** The significant correlation results (*p* < .05, permutation test) of post hoc analysis of canonical correlation analysis (CCA) mode between the degree variate and the clinical variate in the patients with disorders of consciousness (DOC).

|  |  | *r-*value | *p-*value |
| --- | --- | --- | --- |
| Clinical variate | Motor sub-score | 0.52 | 1.46×10^-2^ |
|  | Arousal sub-score | 0.57 | 6.41×10^-3^ |
| Degree variate | *D^i^*^n^ of right insula | -0.57 | 7.35×10^-3^ |
|  | *D*^out^ of the LIPG | -0.48 | 2.73×10^-2^ |
|  | *D*^out^ of the right precentral gyrus | -0.44 | 4.56×10^-2^ |
| arousal sub-score | *D*^in^ of right insula | -0.46 | 3.49×10^-2^ |

Abbreviations: LIPG, left inferior parietal gyrus; *D*^in^, in-degree; *D*^out^, out-degree.

**Supplementary Table 5** The significant correlation results (*p* < .05, permutation test) of post hoc analysis of canonical correlation analysis (CCA) mode between the motif variate and the clinical variate in the patients with disorders of consciousness (DOC).

|  |  | *r-*value | *p-*value |
| --- | --- | --- | --- |
| Clinical variate | Auditory sub-score | 0.78 | 2.74×10^-5^ |
|  | Visual sub-score | 0.72 | 2.44×10^-5^ |
|  | Motor sub-score | 0.66 | 1.08×10^-3^ |
|  | Oromotor sub-score | 0.58 | 5.74×10^-3^ |
| motif variate | MFS of M_4_ within the DMN | 0.55 | 9.13×10^-3^ |
|  | MFS of M_5_ within the DMN | 0.52 | 1.67×10^-2^ |
|  | MFS of M_2_ between the CON-DMN | -0.44 | 4.12×10^-2^ |
|  | MFS of M_2_ between the FPN-versus-all-others RSN | -0.54 | 1.13×10^-2^ |
|  | MFS of M2 between the DMN-versus-all-others RSN | -0.44 | 4.76×10^-2^ |
|  | M2 within the whole brain | -0.74 | 1.40×10^-4^ |
| auditory sub-score | MFS of M_2_ between the FPN-versus-all-other RSNs | -0.46 | 3.41×10^-2^ |
|  | M_2_ within the whole brain | -0.46 | 3.71×10^-2^ |
| visual sub-score | MFS of M_4_ within the DMN | 0.45 | 3.84×10^-2^ |
|  | M_5_ within the DMN | 0.78 | 2.65×10^-5^ |
|  | MFS of M_2_ between the DMN-versus-all-other RSNs | -0.49 | 2.44×10^-2^ |
|  | M_2_ within the whole brain | -0.45 | 4.17×10^-2^ |
| motor sub-score | MFS of M_4_ within the DMN | 0.48 | 2.73×10^-2^ |
|  | MFS of M_2_ within the whole brain | -0.53 | 1.37×10^-2^ |
| oromotor sub-score | MFS of M_5_ within the DMN | 0.44 | 4.41×10^-2^ |
|  | MFS of M_2_ between the DMN-versus-all-other RSNs | -0.48 | 2.73×10^-2^ |

Abbreviations: MFS, motif frequency spectrum; RSN, resting-state network; CON, cingulo-opercular network; DMN, default mode network; FPN, frontoparietal network.

**Supplementary Table 6** Comparison of global efficiency and global clustering coefficient in the whole brain between the patients with disorders of consciousness (DOC) and the healthy controls (HC)

| Different strategies | Metrics | DOC | HC | *t-*value | *p-*value |
| --- | --- | --- | --- | --- | --- |
| No spatial smoothing | Global efficiency | 0.29 | 0.26 | 1.59 | 0.118 |
|  | Global clustering coefficient | 0.23 | 0.26 | -3.83 | 0.0008* |
| Max-lag correlation | Global efficiency | 0.26 | 0.19 | 1.70 | 0.101 |
|  | Global clustering coefficient | 0.24 | 0.27 | -4.08 | 0.001* |

*Note.* * *p* < .05 (permutation test). A positive (negative) *t-*value indicates that the metric was higher (lower) in the patients than in the controls.


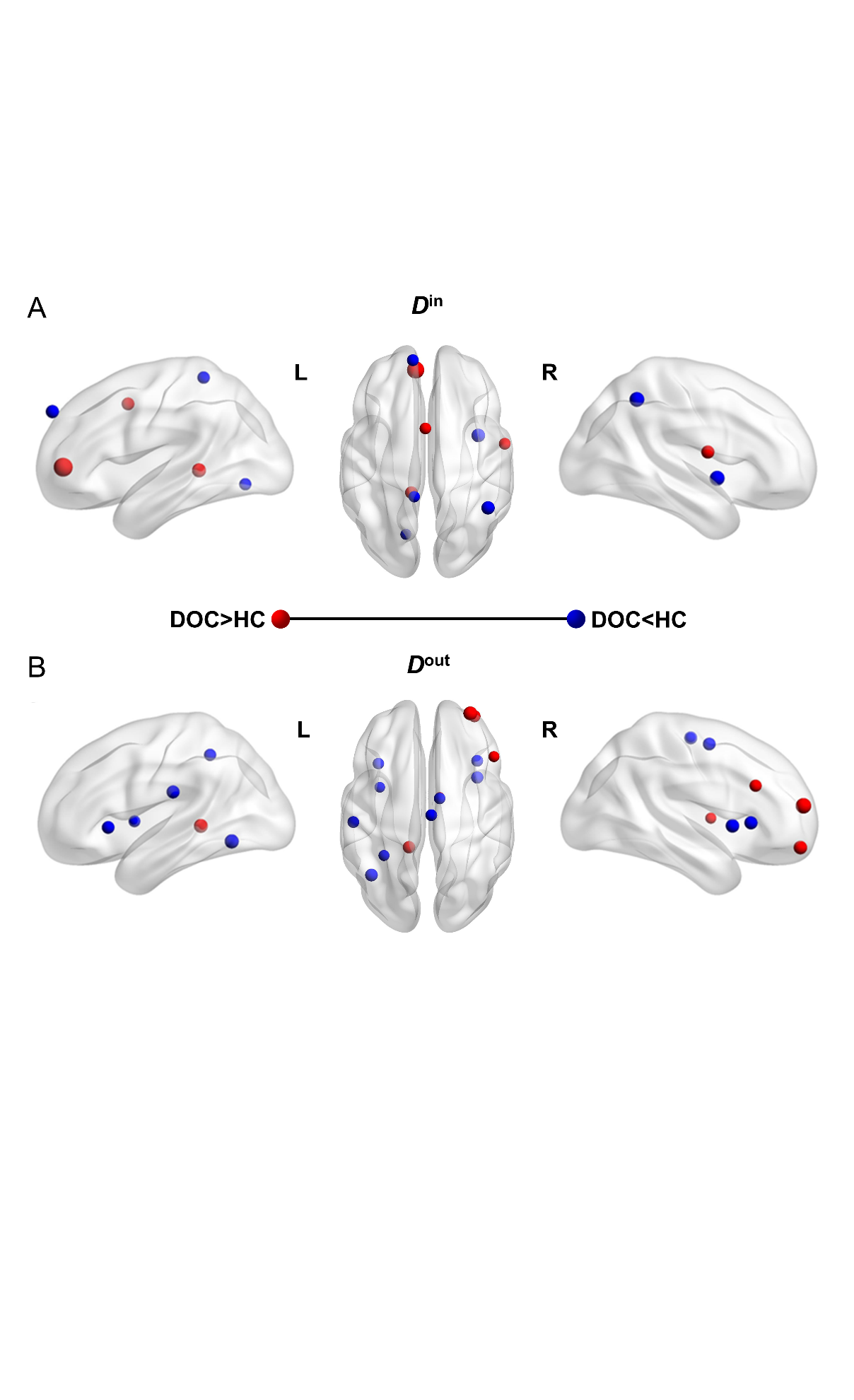


**Supplementary Figure 1** Nodes with significant differences in either in-degree (*D*^in^) or out-degree (*D*^out^) between the patients with disorders of consciousness (DOC, *n* = 21) and the healthy controls (HC, *n* = 21) (*p* < .05, permutation test, FDR-corrected) without spatial smoothing. **(A)** *D*^in^ and **(B)** *D*^out^. The nodes color-coded in red (blue) indicate that *D*^in^ or *D*^out^ was significantly higher (lower) in the patients than in the controls. The size of node is proportional to the absolute value of the *t-*value.


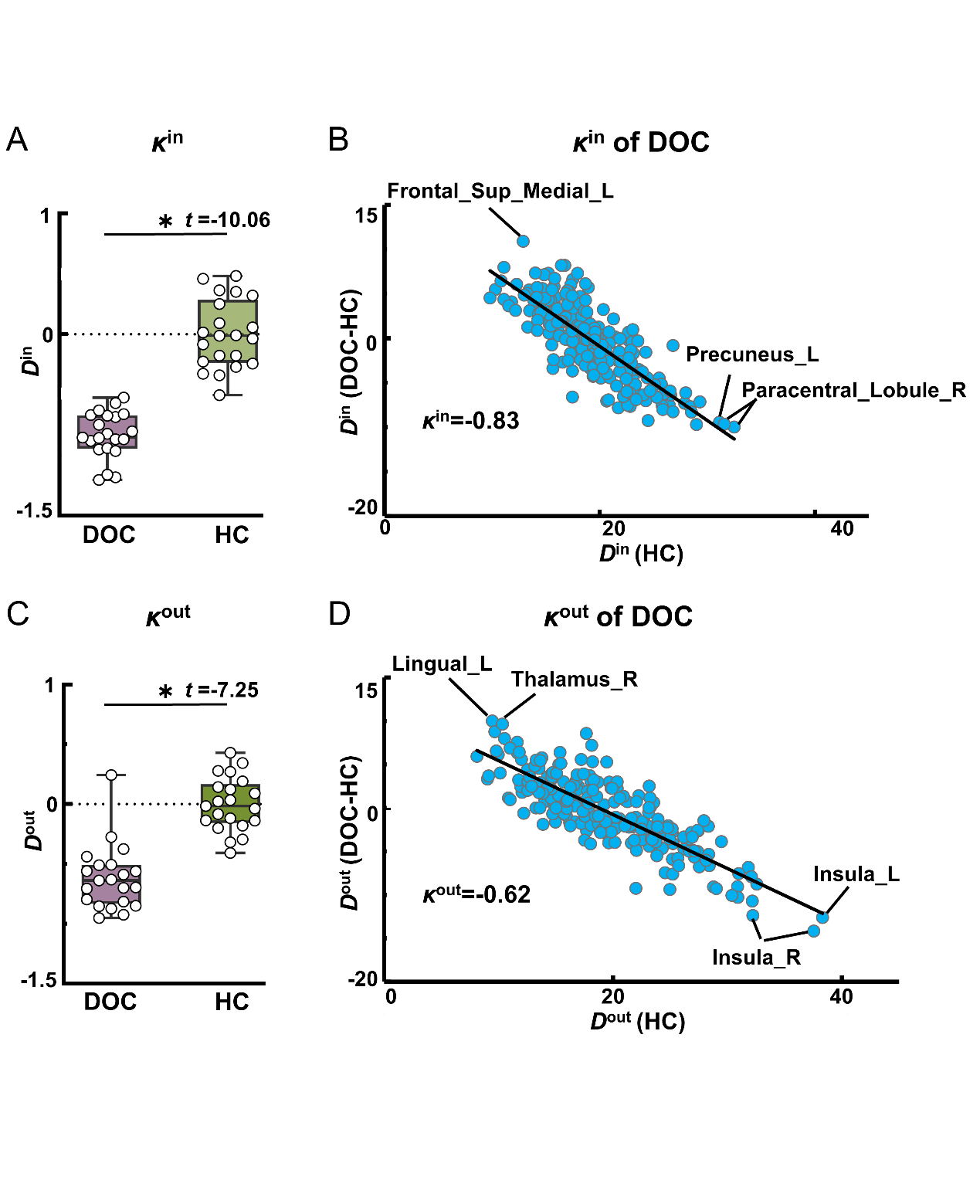


**Supplementary Figure 2** Plots of the hub disruption index for the patients with disorders of consciousness (DOC, *n* = 21) and the healthy controls (HC, *n* = 21) without spatial smoothing. **(A)** Hub disruption index of in-degree (*κ*^in^). The asterisk indicates a significant difference between the patients and the controls (*p* < .05, permutation test). **(B)** *κ*^in^ of the DOC patients. The *x*-axis corresponds to the mean in-degree (*D*^in^) of the controls, and the *y*-axis to the difference between the mean *D*^in^ of the patients and the mean *D*^in^ of the controls at each node. The slope of the fitted straight-line represents the mean *κ*^in^ for the patients. The labeled nodes show the radically altered *D*^in^ in the patients compared with the controls. **(C)** Hub disruption index of out-degree (*κ*^out^). **(D)** *κ*^out^ of the DOC. Abbreviations: Sup, superior; L (R), left (right) hemisphere


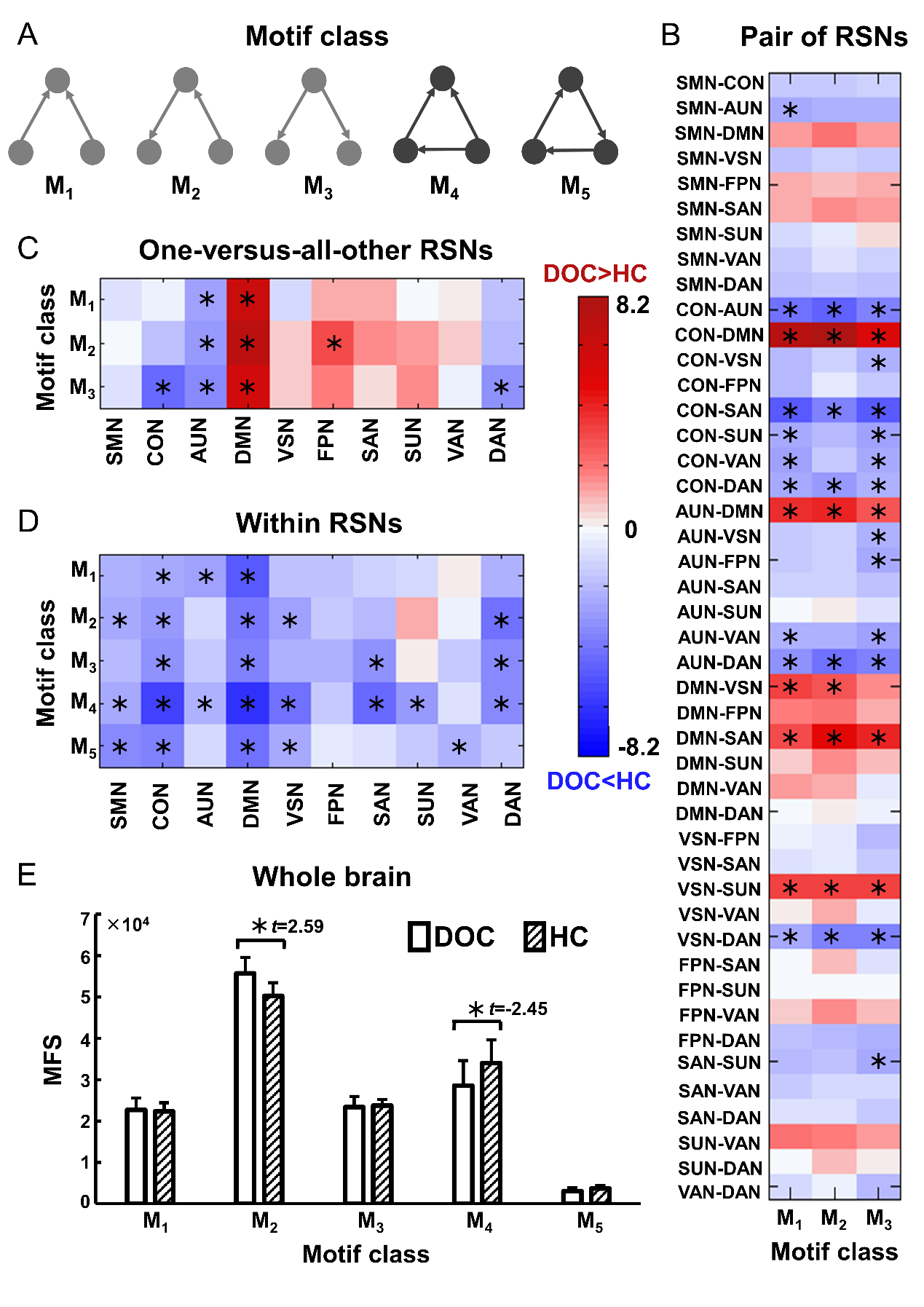


**Supplementary Figure 3** Comparison of the motif frequency spectrum (MFS) between the patients with disorders of consciousness (DOC, *n* = 21) and the healthy controls (HC, *n* = 21) without spatial smoothing. **(A)** The five uni-directional motif classes (M_1_, M_2_, M_3_, M_4_, and M_5_) derived from three nodes. The *t*-value matrix of the MFS for **(B)** between pairs of RSNs, **(C)** between one-versus-all-other RSNs, and **(D)** within RSNs. An asterisk indicates a significant between-group difference (*p* < .05, permutation test, FDR-corrected). **(E)** The mean MFS for the five motif classes in the whole brain.

Abbreviations: SMN, sensorimotor network; CON, cingulo-opercular network; AUN, auditory network; DMN, default mode network; VSN, visual network; FPN, fronto-parietal network; SAN, salience network; SUN, subcortical network; VAN, ventral attention network; DAN, dorsal attention network


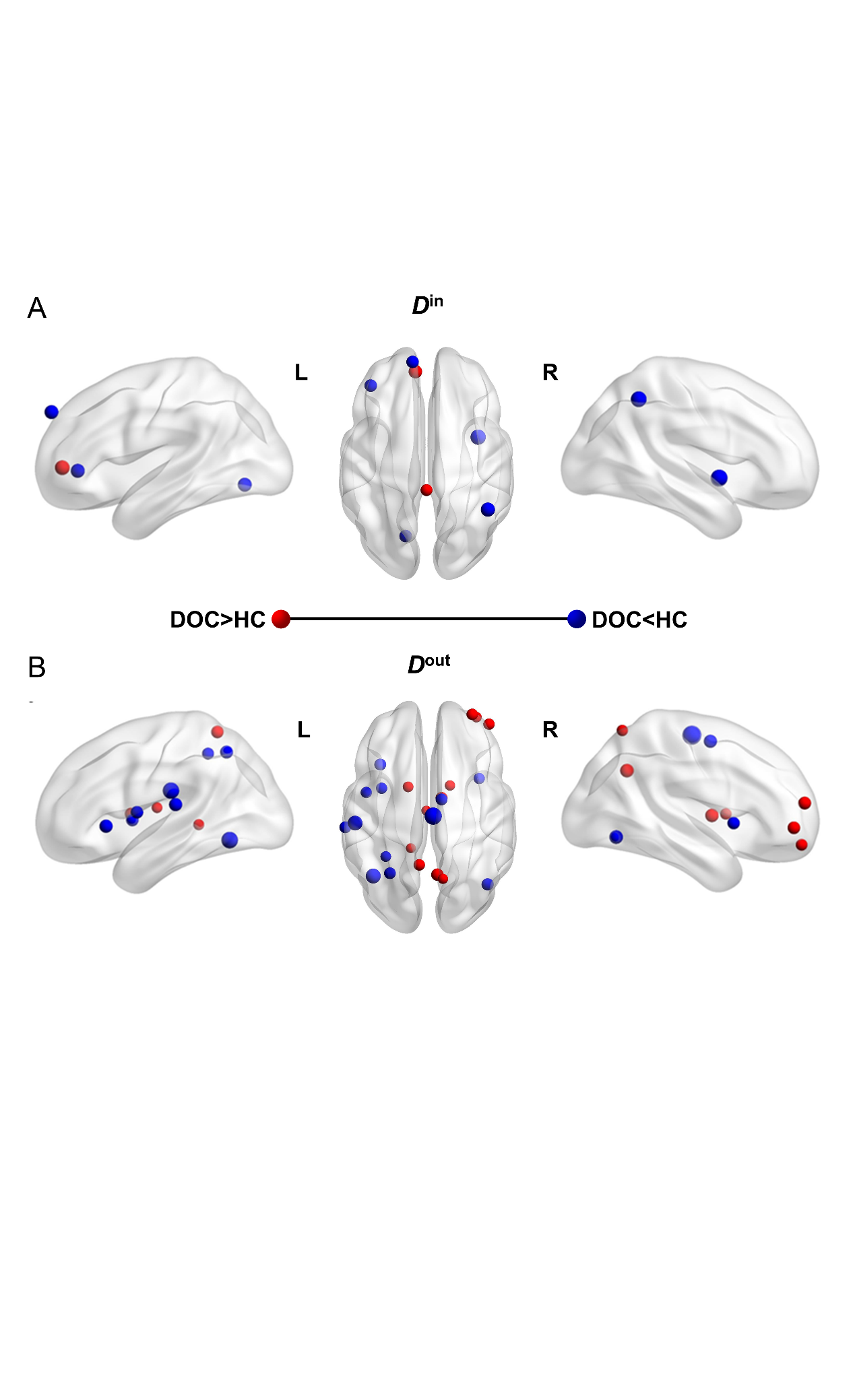


**Supplementary Figure 4** Nodes with significant differences in either in-degree (*D*^in^) or out-degree (*D*^out^) between the patients with disorders of consciousness (DOC, *n* = 21) and the healthy controls (HC, *n* = 21) (*p* < .05, permutation test, FDR-corrected) for max-lag correlation. **(A)** *D*^in^ and **(B)** *D*^out^. The nodes color-coded in red (blue) indicate that *D*^in^ or *D*^out^ was significantly higher (lower) in the patients than in the controls. The size of node is proportional to the absolute value of the *t*-value.

**
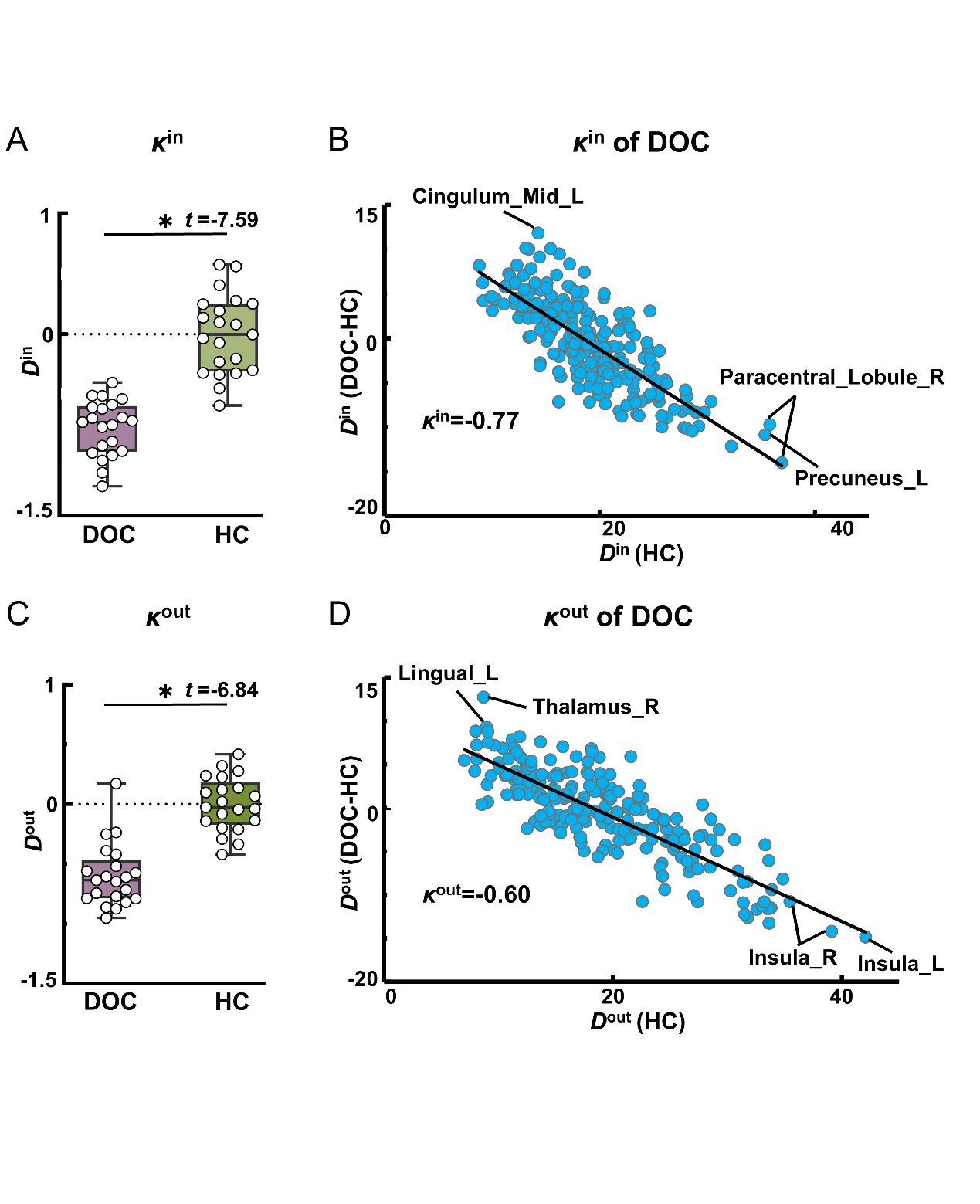
**

**Supplementary Figure 5** Plots of the hub disruption index for the patients with disorders of consciousness (DOC, *n* = 21) and the healthy controls (HC, *n* = 21) for max-lag correlation. **(A)** Hub disruption index of in-degree (*κ*^in^). The asterisk indicates a significant difference between the patients and the controls (*p* < .05, permutation test). **(B)** *κ*^in^ of the DOC. The *x*-axis corresponds to the mean in-degree (*D*^in^) of HC, and the *y*-axis to the difference between mean *D*^in^ of DOC and HC at each node. The slope of the fitted straight-line represents the mean *κ*^in^ for the patients. The labeled nodes show the radically altered *D*^in^ in the patients compared with the controls. **(C)** Hub disruption index of out-degree (*κ*^out^). **(D)** *κ*^out^ of the DOC.

Abbreviations: Mid, middle; L (R), left (right) hemisphere


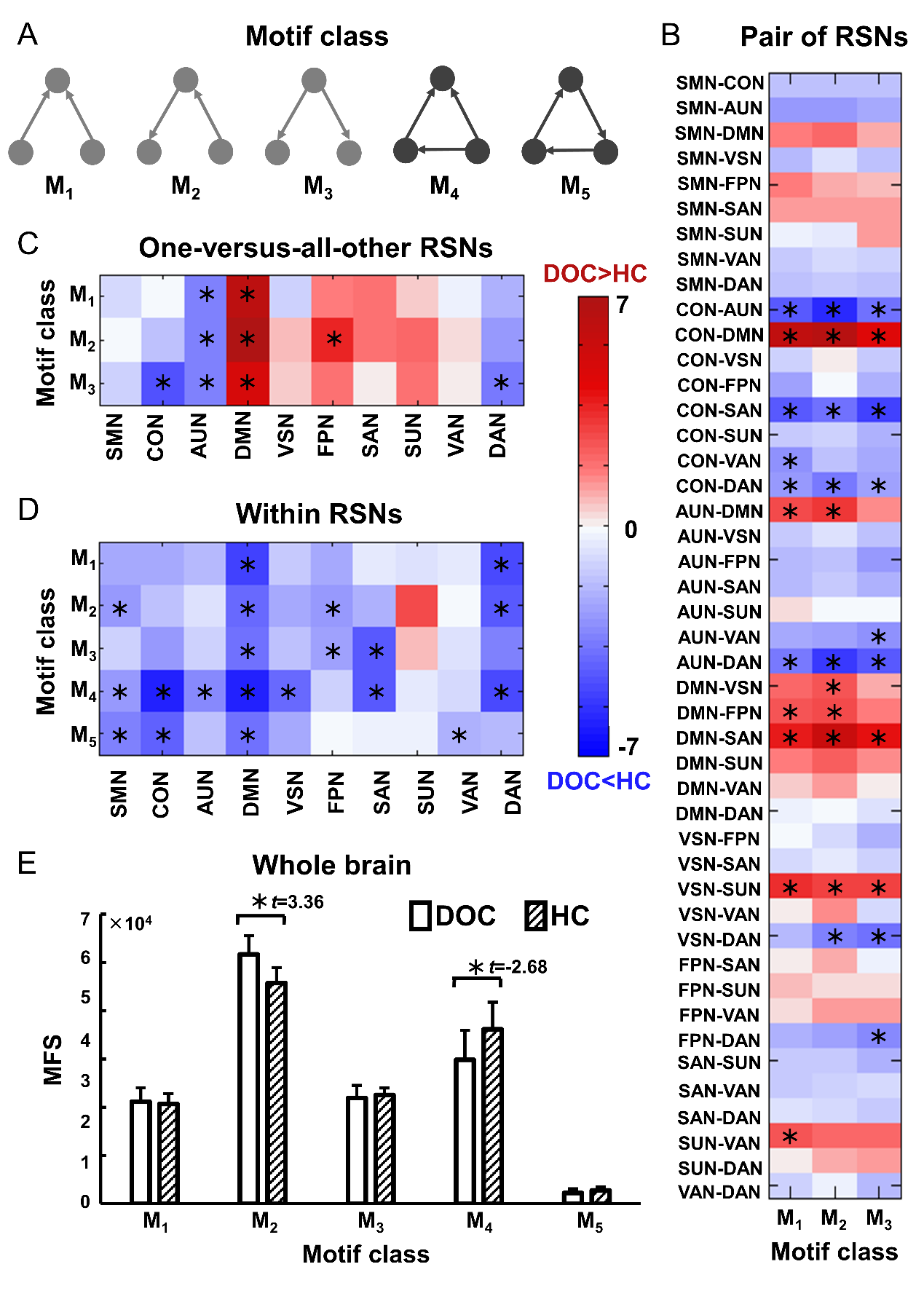


**Supplementary Figure 6** Comparison of the motif frequency spectrum (MFS) between the patients with disorders of consciousness (DOC, *n* = 21) and the healthy controls (HC, *n* = 21) for max-lag correlation. **(A)** The five unidirectional motif classes (M_1_, M_2_, M_3_, M_4_, and M_5_) derived from three nodes. The *t*-value matrix of the MFS for **(B)** between pairs of RSNs, **(C)** between one-versus-all-other RSNs, and **(D)** within RSNs. An asterisk indicates a significant between-group difference (*p* < .05, permutation test, FDR-corrected). **(E)** The mean MFS for the five motif classes in the whole brain.

Abbreviations: SMN, sensorimotor network; CON, cingulo-opercular network; AUN, auditory network; DMN, default mode network; VSN, visual network; FPN, fronto-parietal network; SAN, salience network; SUN, subcortical network; VAN, ventral attention network; DAN, dorsal attention network
